# miR-21 Promotes Fibrogenic Epithelial-to-Mesenchymal Transition of Epicardial Mesothelial Cells Involving Programmed Cell Death 4 and Sprouty-1

**DOI:** 10.1371/journal.pone.0056280

**Published:** 2013-02-18

**Authors:** Hasse Brønnum, Ditte C. Andersen, Mikael Schneider, Maria B. Sandberg, Tilde Eskildsen, Solveig B. Nielsen, Raghu Kalluri, Søren P. Sheikh

**Affiliations:** 1 Laboratory for Molecular and Cellular Cardiology, Department of Clinical Biochemistry and Pharmacology, Odense University Hospital and Department of Cardiovascular and Renal Research, University of Southern Denmark, Odense, Denmark; 2 Division of Matrix Biology, Department of Medicine, Beth Israel Deaconess Medical Center and Harvard Medical School, Boston, Massachusetts, United States of America; Leiden University Medical Center, The Netherlands

## Abstract

The lining of the adult heart contains epicardial mesothelial cells (EMCs) that have the potential to undergo fibrogenic Epithelial-to-Mesenchymal Transition (EMT) during cardiac injury. EMT of EMCs has therefore been suggested to contribute to the heterogeneous fibroblast pool that mediates cardiac fibrosis. However, the molecular basis of this process is poorly understood. Recently, microRNAs (miRNAs) have been shown to regulate a number of sub-cellular events in cardiac disease. Hence, we hypothesized that miRNAs regulate fibrogenic EMT in the adult heart. Indeed pro-fibrogenic stimuli, especially TGF-β, promoted EMT progression in EMC cultures, which resulted in differential expression of numerous miRNAs, especially the pleiotropic miR-21. Accordingly, ectopic expression of miR-21 substantially promoted the fibroblast-like phenotype arising from fibrogenic EMT, whereas an antagonist that targeted miR-21 blocked this effect, as assessed on the E-cadherin/α-smooth muscle actin balance, cell viability, matrix activity, and cell motility, thus making miR-21 a relevant target of EMC-derived fibrosis. Several mRNA targets of miR-21 was differentially regulated during fibrogenic EMT of EMCs and miR-21-dependent targeting of Programmed Cell Death 4 (PDCD4) and Sprouty Homolog 1 (SPRY1) significantly contributed to the development of a fibroblastoid phenotype. However, PDCD4- and SPRY1-targeting was not entirely ascribable to all phenotypic effects from miR-21, underscoring the pleiotropic biological role of miR-21 and the increasing number of recognized miR-21 targets.

## Introduction

Cardiac fibrosis is a prominent element of cardiac disease, and involves numerous biochemical and cellular events leading to interrupted homeostasis of the extracellular matrix, thus ultimately impairing heart performance. The traditional concept that cardiac fibrosis is solely mediated by the activation and differentiation of residing interstitial fibroblasts into myofibroblasts has recently been challenged [1], [2]. In addition to contributions from bone-marrow-derived cells, as much as 35% of the fibroblasts in cardiac fibrosis can originate from Endothelial-to-Mesenchymal Transition (EndMT) of microvascular endothelial cells, as studied in a pressure-overload model [3]. Additionally, there is increasing evidence, not only *in vitro* [4], [5] but also *in vivo* [6], [7], [8], that fibroblast-like cells in the injured adult heart can arise from Epithelial-to-Mesenchymal Transition (EMT) of the epicardial mesothelial cells (EMCs) lining the heart. During cardiac development, the epicardium is formed by migration of mesothelial cells from the proepicardial organ, and subsequently some of the mesothelium generates multipotent epicardium-derived cells (EPDCs). Those EPDCs have the potential to differentiate into all major cell populations of the heart by EMT, including endothelial cells, smooth muscle cells, fibroblasts, and even cardiomyocytes [9]–[14]. In the adult heart, EMT of the epicardial mesothelium is thought to occur by re-activation of the developmental program [5], [7]. However, in the conditions of the injured adult heart, the EMT of EMCs predominantly generates fibroblast-like cells, thereby contributing to the default repair-driven fibrotic response [8], [15]. Nonetheless, the adult EMCs or multipotent EPDCs seems to withstand their inherent progenitor potential if not challenged by the fibrogenic environment of the injured myocardium [8], and inhibiting fibroblast-committed EMT to facilitate myogenic/angiogenic differentiation is of increasing interest within the field of regenerative tissue engineering.

Cardiac disease and development is substantially regulated by microRNAs (miRNAs), which are small non-coding RNAs that post-transcriptionally regulate gene expression [16]. Therefore, miRNAs have been suggested as clinically relevant targets of cardiac disease [17], [18], [19], [20]. In specific, miR-21 and −29 have been identified as key players in cardiac remodeling by affecting interstitial fibroblasts [21], [22], [23]. Although miRNAs primarily are believed to fine-tune biological processes, the impact of these miRNAs exert quite substantial effects on the cellular level, and anti-miR-21 treatment may revert *in vivo* cardiac fibrosis as shown in mouse models [22]. Additionally, miRNAs have been implicated as major modulators of EMT in cancer cell lines [24]–[28] as well as implicated in cardiac EndMT [29], but their involvement in cardiac EMT remains to be determined. We therefore set forth to determine the role of miRNAs in fibrogenic EMT of adult EMCs. We hereby show that cardiac fibrogenic EMT, and the associated generation of fibroblast-like cells, is indeed significantly modulated by miRNAs, especially miR-21 through direct targets including Programmed Cell Death 4 (PDCD4) and Sprouty homolog 1 (SPRY1). This suggests that miR-21 is a potential therapeutic target for manipulation of EPDC fate-decision.

## Methods

### Ethics Statement

All experiments involving cell cultures were performed with tissue from adult (8–10 weeks) Sprague-Dawley rats (Taconic Europe, Denmark) that were sacrificed by CO2 and cervical dislocation. Cell culture experiments were performed in accordance with §1 in the Danish proclamation of law on animal experimentation (LBK No. 1306, 23/11/2007). Transverse aortic constriction (TAC) and left anterior descending artery (LAD) ligation was performed on 12-week old female C57/BL6 mice, which were anesthetized IP with Ketamine (100 mg/kg) and Xylazine (5 mg/kg) and ventilated by tracheal intubation and connection to a MiniVent (Harvard Apparatus) set to a tidal volume 0.2 ml and a frequency of 100 min^−1^. Animal experiments were approved by the Danish National Animal Experiment Inspectorate (Permission # 2009/561-1663 (TAC) and # 2011/561-1966 (LAD ligation).

### EMC Isolation and Culture

**Figure 1:**
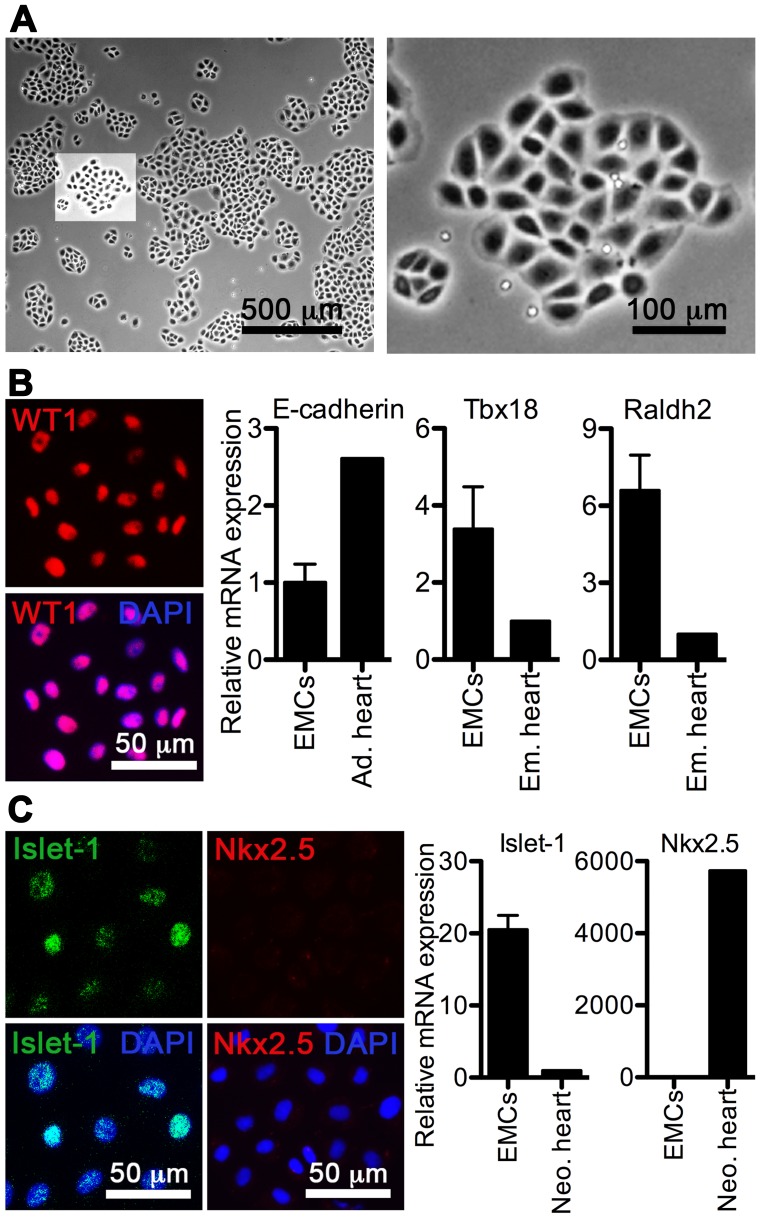
Clonogenic cultures of adult rat EMCs are homogenous with respect to epicardial marker expression. **A**, Phase pictures of isolated and expanded EMCs in culture (500 µm and 100 µm scale, respectively). **B** and **C**, Expression of epicardial markers (Wilms Tumor protein homolog (WT1), E-cadherin, Tbx18, Raldh2, and Islet-1) and the cardiomyogene marker Nkx2.5 in cultured EMCs. Relative qRT-PCR data (means+SD, n = 3) were normalized against GAPDH and RPL13A, and depicted relative to an adult (Ad.), embryonic (Em.), or neonatal (Neo.) rat heart. Rat ventricular fibroblasts were used as negative controls for immunocytochemistry (data not shown).

Dissected rat hearts were washed in a cardioplegic preparation buffer (1.2 mmol/L KH_2_PO_4_ (pH 7.4); 2.4 mmol/L Na_2_CO_3_; 0.11 mol/L NaCl; 2.6 mmol/L KCl; 1.2 mmol/L Mg_2_SO_4_; 11 mmol/L glucose; incubated at 37°C, 5% CO_2_, 24 h before use) supplemented with heparin. Isolated ventricles were cut into three pieces, washed twice with preparation buffer/50 IE/ml heparin, and sequentially digested (2×12****min., stirring at RT) with 0.15% Trypsin (BD Difco) in preparation buffer supplemented with 0.01% DNase I (Sigma-Aldrich). Dissociated cells were harvested by centrifugation 7 min., 300×g, 4°C. Red blood cells were lysed (168 mmol/L NH_4_Cl; 10 mmol/L NaHCO_3_; 0.1 mmol/L Tetrasodium EDTA) prior to wash, and remaining cells were resuspended in growth medium (Dulbecco’s Modified Eagles Medium (DMEM; BE12-604F/U1, Lonza)/10% FBS (Gibco; 10270-106)/1% Penicillin-streptomycin (Gibco). Isolated cells were plated in growth medium onto polystyrene dishes, allowed to attach for 1****h, and washed twice in growth medium. At day 10 of culture, homogeneous, non-differentiated clones comprising 100–150 cells (Figure 1A) were isolated using cloning cylinders (Sigma-Aldrich) and 0.25% Trypsin-EDTA (Invitrogen). Clones derived from the same rat heart were pooled, plated at 3–5 cells/cm^2^ in polystyrene dishes, and cultured for additional 33 days with one intervening passage, and medium change every fourth day. EMCs were isolated individually from 8 animals, frozen in liquid nitrogen until use, then gently thawed, cultured and used in passage P5–P6 for all experiments. EMT was induced in ∼20% confluent EMC cultures by incubation with 10 ng/mL of recombinant human IL-1β, TNF-α, or TGF-β1 (all R&D Biosystems) in DMEM/2% FBS for 48****h and then with 10 ng/mL IL-1β, TNF-α, or TGF-β1 in DMEM/10% FBS for further 48****h.

### Transverse Aortic Constriction

Under sterile conditions the chest was opened at the second intercostal space and the thymus glands were superiorly reflected. The transverse thoracic aortic branch was dissected and a 7–0 Prolene suture was tied around the aorta between right and left common carotid artery against a blunted 26-gauge needle. Hearts were obtained at day 1, 4, 7, 14, and 21 after TAC and miR-21 profiling was conducted on biological triplicates. A time-matched control group (also n = 3) underwent a sham operation, thus excluding TAC.

### Left Anterior Descending Artery Ligation

Under sterile conditions the chest was opened at the fourth intercostal space and the LAD dissected. Acute myocardial infarction was induced by LAD ligation using a 7-0 prolene suture. Hearts were obtained at day 2, 4, 7, 14 and 28 after LAD ligation, and miR-21 profiling was performed on two, four, two, one and four pooled hearts, respectively. The time-matched control group underwent a sham operation and miR-21 profiling was performed on one or two pooled hearts.

### Immunocytochemistry

Cells were fixed with 4% paraformaldehyde and otherwise prepared as previously described [30]. Specific for SPRY1 stainings, cells were fixed with ice cold methanol. Cells were incubated overnight with primary antibodies (see below), which were visualized using Alexa 488 or 555 conjugated secondary antibodies (diluted 1∶200). Non-specific staining by antibodies was identified by mouse IgG2a, IgG1 or rabbit IgG isotype controls or, in case of Islet1, WT1 or Nkx2.5, rat ventricular fibroblasts [30] were used as negative controls. Nuclei were counterstained with DAPI (Vectorshield, Vector Laboratories, UK), and images were acquired using a Leica DMI4000B Cool Fluo Package instrument equipped with a Leica DFC340 FX Digital Cam. In all experiments, exposure (camera settings) and picture processing (brief adjustment of contrast/brightness and color balance by Photoshop (Version 10.0.1)) were applied equally to the entire image from sample sections and controls (mouse isotypes, rabbit IgG, and rat ventricular fibroblasts).

### Antibodies

Rabbit anti-WT1 (0.035 µg/ml, Abcam, Cambridge, UK, ab33821), mouse anti-Islet1 (13 µg/ml, Developmental Studies Hybridoma Bank, clone 39.4D5), rabbit anti-Nkx2.5 (4 µg/ml, Santa Cruz Biotechnology, #sc-14033), mouse anti-CD45 (20 µg/ml, BD Biosciences, clone OX-1), mouse anti-Troponin T (1.6 µg/ml, Developmental Studies Hybridoma Bank, clone CT3), mouse anti-α-SMA (11 µg/ml, Sigma-Aldrich, clone 1A4), rabbit anti-S100A4 (0.38 µg/ml, kind gift from Dr. Eugene Lukanidin, Danish Cancer Society, Copenhagen, Denmark), rabbit anti-PDCD4 (1 µg/ml, Cell Signaling, D29C6), rabbit anti-SPRY1 (0.67 µg/ml, Santa Cruz, #sc-30048), mouse anti-IgG2a,κ (Sigma-Aldrich, UPC-10), mouse anti-IgG1,κ (Sigma-Aldrich, MOPC-21), rabbit anti-IgG (Santa Cruz, #sc-2027), Alexa Fluor donkey anti-mouse 555, Alexa Fluor donkey anti-mouse 488, Alexa Fluor donkey anti-goat 488, Alexa Fluor donkey anti-goat 488, Alexa Fluor donkey anti-rabbit 555, Alexa Fluor donkey anti-rabbit 488, (Molecular Probes, Invitrogen, DK). Antibodies for CD31 flow cytometry; PE-mouse anti-rat CD31 (0.125 µg/100 µl/387,500 cells, BD Pharmingen, clone TLD.3A12) and PE-mouse IgG1,κ Isotype Control (BD Pharmingen, clone MOPC-31c).

### Flow Cytometry

Flow cytometry for CD31 was performed as previously reported. The adipose derived stromal vascular fraction, rich in endothelial cells, was isolated [30] from adult WK rats and used as a positive control.

### Transfections

EMCs were equilibrated in serum-free medium for 1****h and subsequently transfected for 4****h with pre-miRs, anti-miRs or siRNAs using the Lipofectamine 2000 protocol (Invitrogen, DK) as recommended by the manufacturer, except that DMEM was applied as transfection medium. Optimal transfections were achieved at 20 nM probe (Figs. S4 and S7), and this concentration was used for all experiments. EMCs transfected with pre-miRs were washed and further cultured for 48****h in DMEM/2% FBS until analysis. For single transfection with anti-miRs or in combination with siRNAs, EMCs were pre-incubated in DMEM/2% FBS supplemented with 10 ng/mL recombinant human TGF-β1 (R&D Biosystems) to initiate EMT. After 20****h, cultures were transfected with siRNAs and/or anti-miRs for 4****h and subsequently incubated with 10 ng/mL recombinant human TGF-β1 for another 24****h. Pre-miR-21 (PM10206) and pre-miR scramble controls (with [AM17212] or without [AM17110] FAM) were from Ambion, Denmark. The applied anti-miRs were designed in accordance with previously validated Locked nucleic acid-based anti-miRs [31] (Scramble, 5′-+T*G*+T*A*A*+C*+A*C*G*+T*C*+T*A*+T*+A-3′; anti-miR-21, 5′-+T*C*+A*G*T*+C*+T*G*A*+T*A*+A*G*+C*+T 3′;+denotes LNA base, *denotes phosphorothioate bond) and were purchased from Exiqon, Denmark.

For siRNA transfections, the Silencer Select system from Applied Biosystems was used. Optimal knock-down was achieved by assessing three variants of each siRNA (PDCD4-siRNA (4390771; s133733; s133734; s133735) and SPRY1-siRNA (439071; s148913; s235122; s235123)). Efficient mRNA knockdowns (>75%) relatively to a negative siRNA control (4390846) were reached with s133735 (PDCD4) and s235123 (SPRY1) (Fig. S6), and all further experiments were therefore performed with these siRNA variants.

### Cell Number and Volume

Cultured cells were gently detached by 0.25% Trypsin-EDTA (Invitrogen, Denmark). The cell number and cell diameter were determined using a Beckman Coulter Counter Z2 (Ramcon, Denmark) fitted with a 100 µm aperture. The size range of particles counted was set at 10–25 µm and counting was performed in three independent experiments, each comprising triplicate measurements.

### MTT Assay

Viability was validated by a standard Vybrant MTT cell proliferation assay (Molecular Probes, Invitrogen), measuring the mitochondrial activity.

### Wound Healing Assay

EMCs were cultured to ∼90% confluence in a 12-wells plate and wound healing was assessed as previously described [32]. In brief, scratches were induced mechanically with a pipette tip. The wells were subsequently washed twice and incubated for 21****h. The wound widths were measured at three pre-defined spots in wells at both 0****h and 21****h after scratch induction. The relative wound size was computed as the relative decrease in the wound width across time.

### ELISA

TIMP-1 amounts in EMC culture supernatants were measured by a rat TIMP-1 sandwich ELISA kit (R&D Biosystems, DY580) as recommended by the manufacturer. The specific amount (pg/cell) of TIMP-1 was calculated by standardizing with the cell numbers as quantified by Coulter counting.

### Western Blotting

Western blotting for PDCD4 and SPRY1 was performed using the NuPAGE electrophoresis system (Invitrogen) as previously described [33]. Reduced protein lysates were separated on a 4–12% Bis-Tris gradient gel in MOPS running buffer and blotted onto a PVDF membrane. Primary antibodies were used at a concentration of 0.34 µg/ml for rabbit anti-PDCD4 and 2 µg/ml for rabbit anti-SPRY1. HRP-conjugated swine anti-rabbit immunoglobulins (Dako, P0217) was used as secondary antibody and bands were visualized by chemiluminescense with an ECL kit (GE Healthcare). Band intensities were quantified using ImageJ 1.43 software and presented relative to the total protein amount, as estimated by Coomassie staining.

### RNA Extraction

Total RNA was extracted with TriReagent (Molecular Research Center, Inc., Cincinnati, OH), as previously described [30]. RNA purity and stability was validated by Nanodrop (Saveen Werner, Denmark) measurements and capillary electrophoresis on an Agilent 2100 BioAnalyzer using RNA Nano Chips (Agilent technologies, Denmark).

### Relative qRT-PCR

Relative qRT-PCR was performed on 400 ng reverse-transcribed cDNA (High Capacity cDNA RT kit; Applied Biosystems, Denmark) using SYBR green (Appiled Biosystems) as reporter dye and 10 µmol/L rat-specific primers. All primers were from DNA-Technology A/S, Denmark (Table S1). As recommended by others [34], [35], and as previously described [30], we used the qBase^+^ software to normalize all qRT-PCR data against multiple stably expressed reference genes (mRNAs or miRNAs) as determined by the geNorm platform (Table S2). TaqMan® microRNA Assays (Applied Biosystems) were applied for expression analyses of miR-21 (Assay ID 0397) and the endogenous controls let-7f (Assay ID 0382), miR-17-5p (Assay ID 2308), and miR-195 (Assay ID 0494).

### miRNA Microarray Analysis

miRNA expression profiling was performed as two-color common reference hybridizations on LNA-based arrays (miRCURY LNA™ microRNA Array ready-to-spot probe set, v.10, 208110-A, Exiqon, Denmark) spotted in-house on CodeLink™ HD Activated slides (DHD1-0023, SurModics, Eden Prairie, MN) according to the manufacturer’s protocol. 2×250 ng total RNA was used for each hybridization. Samples were labeled with Hy3 and the common reference (pool of all samples) was labeled with Hy5, by use of miRCURY LNA microRNA Array Power labeling kit (208032-A, Exiqon) and hybridized for 16 hours. Slides were washed (208021, Exiqon) and scanned on an Agilent (G2565CA) Microarray Scanner, and scans were analyzed by GenePix 6.0 software. Normalization and background correction was performed in R using “vsn” package from Bioconductor, and triplicate spots were averaged. Differential expression was next assayed using the “limma” package also from Bioconductor by fitting to the eBayes linear model and contrasting individual treatments with untreated controls. Log fold changes (FC) were calculated using the “topTable” function of the limma package. The most regulated miRNAs (−2<FC>2) were presented as log_2_FC in heatmap, and data were hierachially clustered for both sample treatment (IL-1β, TNF-α or TGF-β) and miRNA using Pearson Correlation in the software program MeV 4.5. The data discussed in this publication have been deposited in NCBI's Gene Expression Omnibus (Edgar *et al*., 2002) and are accessible through GEO Series accession number GSE37627 (http://www.ncbi.nlm.nih.gov/geo/query/acc.cgi?acc=GSE37627).

### Statistical Analysis

Data are presented as means±SD from 3 independent experiments, and statistical significance (α = 0.05) was tested by two-tailed t-tests, one-way ANOVA with Tukey’s post test, or two-way ANOVA using GraphPad Prism 5.0a. Data acquired by qRT-PCR were Log2-transformed before statistical analyses.

## Results

### Primary EMC Cultures can be Clonally Obtained

Isolation and cultivation of adult EMCs have largely been performed by explant cultures [6], [7], [15], [36]. To achieve high purity and clone integrity of EMCs, we instead established clonogenic cultures directly isolated from the epicardium. In this manner we obtained homogenous clones exhibiting tight cobblestone morphology, and this epithelioid phenotype was maintained for up to 12 passages (Fig. 1A). EMCs were positive for epicardial markers Wilms Tumor protein homolog (WT1), E-cadherin, Tbx18, Raldh2, and Islet-1, but negative for Nkx2.5 (Fig. 1B–C), which both confirmed their epicardial origin and EMC progenitor commitment [37]. The near 100% purity of EMCs was validated by analyses for CD45, Troponin T, α-smooth muscle actin (α-SMA) and CD31 (Fig. S1), which ruled out contamination from leukocytes, myocytes, smooth muscle cells and endothelial cells.

### EMCs Undergo Fibrogenic EMT *in vitro*

**Figure 2:**
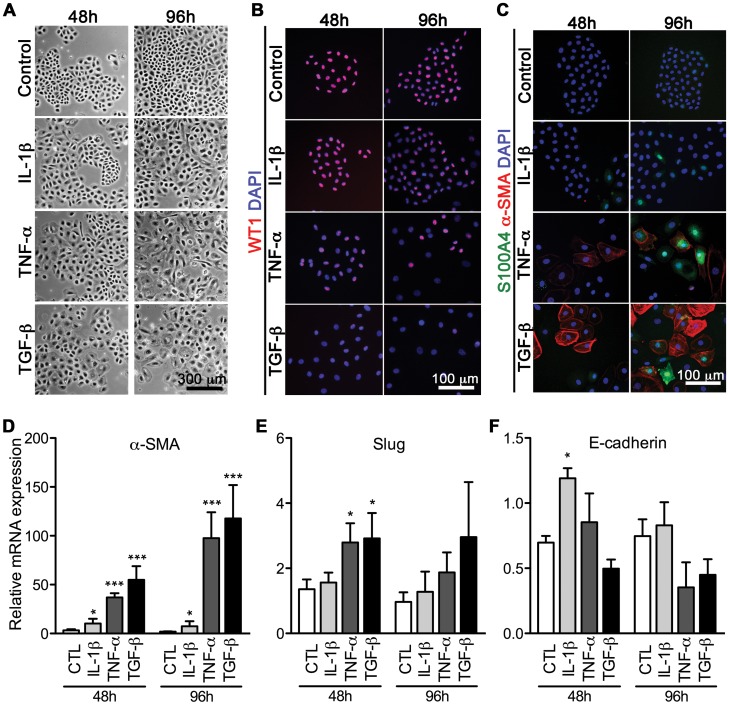
Fibrogenic EMT of EMCs is induced differentially by IL-1β, TNF-α, and TGF-β. Non-confluent EMC cultures were stimulated with IL-1β, TNF-α, or TGF-β, and examined after 48 and 96****h by **A**, phase contrast microscopy for morphology, **B**, immunofluorescence of epicardial cell markers (Wilms Tumor protein homolog (WT1)) and **C**, fibrogenic markers (S100A4 and α-smooth muscle actin (α-SMA)). qRT-PCR for expression of the EMT biomarkers **D**, α-SMA, **E**, Slug and **F,** E-cadherin. For immunofluorescence, fibroblasts and antibody isotype controls (not shown) were used to assess non-specific staining. qRT-PCR data (means+SD, n = 3) were normalized against GAPDH and RPL13A, and statistical significance was tested by one-way ANOVA and treatment effects by Tukey’s post test. *P<0.05, ***P<0.001 vs. the control (CTL) at indicated time-points.

We next asked if the adult EMCs were able to adapt a mesenchymal fibroblast-like cell phenotype by EMT, in accordance with previous reports on cardiac EMT [4], [5], [6], [7], [8]. EMT of epithelial cells including the mesothelium, can be induced by pro-inflammatory cytokines or growth factors, such as TGF-β [38]. To increase experimental specificity and scientific power, we both tested IL-1β, TNF-α, and TGF-β for their efficacy to induce EMT in EMCs (Fig. 2). Indeed, over time, treated EMCs developed a fibroblastoid morphology (Fig. 2A) with loss of the epicardial marker WT1 (Fig. 2B) and up-regulation of mesenchymal markers S100A4 and α-SMA (Fig. 2C), the latter showing fibroblastoid-characteristic stress fiber formation. We validated this by qRT-PCR for α-SMA (Fig. 2D), showing maximal induction after 96****h with a mean fold increase of 8, 98, and 118 for IL-1β, TNF-α, and TGF-β, respectively. As such, the α-SMA expression analysis substantiated the differential ability to induce a mesenchymal phenotype, the potency being TGF-β>TNF-α>>IL-1β.

Classic transcriptional regulators of EMT include Twist-1, Snail and Slug, which all act as repressors of E-cadherin. Accordingly, we found Slug to be significantly up-regulated (approximately 3-fold) after 48****h with TNF-α or TGF-β stimulation (Fig. 2E), while differential loss of E-cadherin was less prominent (Fig. 2F). However, no significant changes were detected in Twist-1 and Snail mRNA levels (results not shown). This may however be explained by the relative high basal levels of Twist-1 and Snail in EMCs (Fig. S2), which is in agreement with a recent report on TGF-β-treated human pulmonary valve progenitor cells [39].

Additionally, we performed phenotypic measurements over time in the IL-1β-, TNF-α- or TGF-β stimulated EMCs (Fig. S3). In accordance with EMT progression [40], we measured a 70% reduction in cell number after 96****h of TGF-β incubation (Fig. S3A and S3C) and a parallel 20% increase in cell volumes for TNF-α and TGF-β treated EMCs (Fig. S3B). We also found a 4-fold induction of TIMP-1 (Tissue inhibitor of metalloproteinase 1) after TGF-β incubation (Fig. S3D). Taken together, this comprehensive analysis of EMT biomarkers showed that *in vitro* cultures of EMCs undergo EMT in response to different inducers, with the potency being TGF-β1> TNF-α>>IL-1β.

### miR-21 is the Major Up-regulated miRNA during EMT

**Figure 3:**
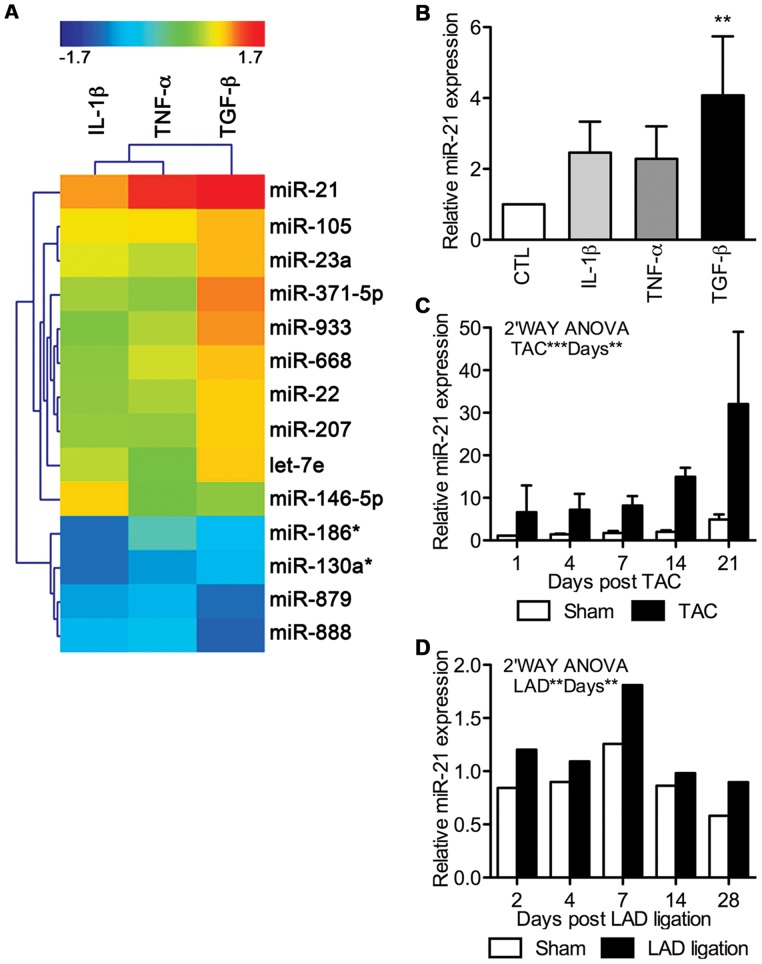
miR-21 is highly up-regulated during fibrogenic EMT. Differentially expressed miRNAs were assessed in EMC cultures stimulated for 48****h with IL-1β, TNF-α, or TGF-β. **A**, Cluster analysis and heatmap of regulated miRNAs (fold change (FC) above 2 or under −2) as detected by microarrays and compared to non-stimulated control cells. **B**, Validation of miR-21 expression after 48****h of EMT induction in EMCs by qRT-PCR (means+SD, n = 3; standardized to the control (CTL)). Statistical significance was tested by one-way ANOVA and treatment effects by Tukey’s post test. miR-21 up-regulation was further validated by qRT-PCR *in vivo* using both a model of **C**, pressure-overload (transverse aortic constriction; TAC) and **D**, myocardial infarction (ligation of left anterior descending artery; LAD). These data were acquired on biological triplicates (means+SD) or a pool of 1–4 hearts, respectively, and analyzed against sham treated animals by two-way ANOVA. All miRNA qRT-PCR data were normalized against miR-17 and miR-195. **P<0.01, ***P<0.001 vs. control.

To assess which miRNAs are implicated in cardiac fibrogenic EMT, we performed miRNA microarray profiling of EMCs treated for 48****h with IL-1β, TNF-α, and TGF-β. As expected from the observed differential power of fibrogenic EMT induction, each of the three different treatments resulted in a distinct miRNA fingerprint (Table S2). More importantly, miR-21 was by far the most up-regulated miRNA in all the treatments (Fig. 3A). We found this novel regulation of miR-21 in cardiac EMT highly remarkable, as miR-21 has been extensively implicated in cardiac disease [17], [18], [19], [23], [41], [42], [43]. The up-regulation of miR-21 was validated by qRT-PCR (Fig. 3B), with TGF-β again being the most potent inducer, and we therefore decided to focus on TGF-β-induced EMT in the remaining part of the study. Interestingly, when testing if the array data had significance *in vivo*, we found a time-dependent miR-21 up-regulation (Fig. 3C, 3D) in two different models of cardiac remodeling in which EMT of EMCs is known to contribute [8], namely both TAC (Fig. 3C) and LAD ligation (Fig. 3D). Before miR-21 profiling, the TAC and LAD ligation models were validated for development of cardiac remodeling, as verified by elevated ANP, BNP, α-SMA and TGF-β expression over time (results not shown). Overall, the results from the miRNA array (Fig. 3A, 3B) combined with the data from the TAC (Fig. 3C) and the LAD ligation (Fig. 3D) model, underscored miR-21 as a major candidate of fibrogenic modulation in the heart, including EMT in EMCs.

### miR-21 Alone Promotes Fibrogenic EMT

We next asked if the robust miR-21 up-regulation also had a functional effect on the development of the mesenchymal, fibroblast-like phenotype generated by fibrogenic EMT. We first over-expressed miR-21 (“Pre-miR-21”) in non-stimulated EMCs, to test if miR-21 alone was able to induce the mesenchymal cell characteristics. Initially, qRT-PCR for miR-21 showed that pre-miR-21 transfected cells expressed 120±31 fold more miR-21 than the control (scramble transfected cells) (Table 1) verifying robust over-expression. We then examined EMT biomarkers in miR-21 over-expressing cells, and found that miR-21 had a mesenchymal promoting effect, highly similar to that observed during TGF-β-induced EMT (Table 1). Accordingly, we found a reduction in E-cadherin expression (−30%) and cell number (−40 to −13%), while α-SMA expression (2 to 22-fold), TIMP-1 production (+40 to +230%), cell volume (+4 to +8%) and motility (+23 to +45%) were all increased (Table 1 and Fig. S5). To confirm these data, we additionally knocked down endogenous miR-21 using an antagonist (“α-miR-21”) during TGF-β-induced EMT. As expected, these experiments clearly showed that development of the mesenchymal phenotype was indeed inhibited by the loss of miR-21 (Table 1). Together these data suggest that miR-21 plays a major role in promoting the phenotypic shift observed during EMT of EMCs.

**Table 1:** miR-21 promotes a mesenchymal phenotype during TGF-β-induced EMT.

	EMT	Transfection	
	TGF-β	Pre-miR-21	TGF-β+α-miR-21
miR-21 expression	4.01±1.7 *	120±31***	0.793±0.050*
E-cadherin expression	0.681±0.10*	0.683±0.081*	2.26±0.34*
α-SMA expression	22±15*	1.83±0.14**	0.513±0.14*
Soluble TIMP-1	2.29±0.47*	1.38±0.088*	0.695±0.033**
Cell number	0.600±0.033**	0.873±0.020**	1.05±0.020*
Mean cell diameter	1.08±0.035	1.04±0.013*	0.996±0.0051
Relative wound size	0.547±0.031**	0.771±0.081*	1.32±0.079*

Biomarkers were evaluated after TGF-β-induced EMT (left column) and compared to effects of transfection (right columns) with either miR-21 agonist in resting EMCs (“Pre-miR-21) or with miR-21 antagonist during TGF-β-induced EMT (“α-miR-21”). All biomarkers are presented relative to control treatments (medium (first column), pre-miR scramble (second column), anti-miR scramble (third column). Representative pictures used for assessment of cell motility (wound size) are in Figure S5.

* *P*<0.05,

** *P*<0.01 as tested by a two-tailed t-test.

### PDCD4 and SPRY1 are Targets of miR-21 in Fibrogenic EMT

**Figure 4:**
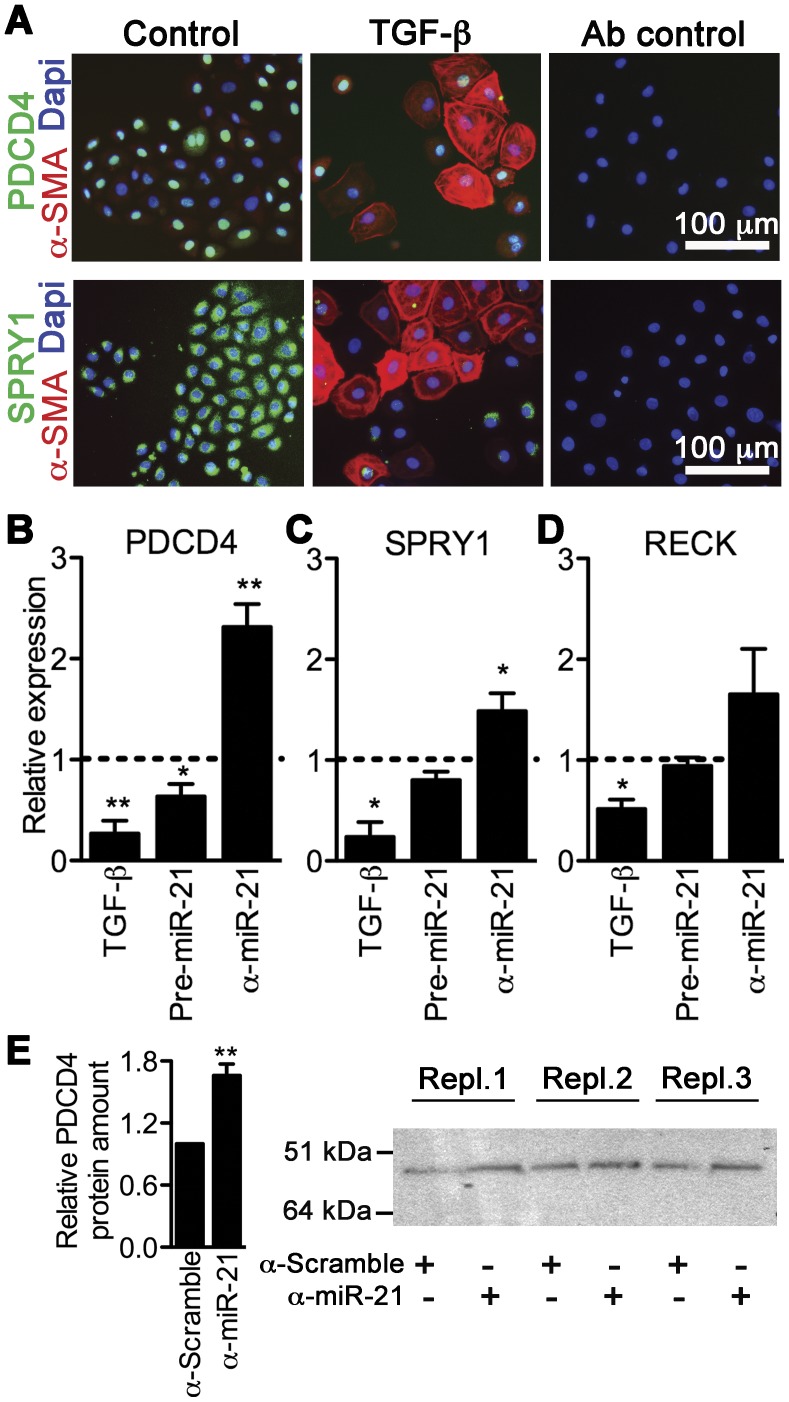
PDCD4 and SPRY1 are miR-21 targets in TGF-β-mediated EMT of EMCs. **A**, TGF-β stimulated EMC cultures were examined after 48 h for expression of PDCD4/α-SMA or SPRY1/α-SMA by immunofluorescence. qRT-PCR detection of PDCD4 (**B**), SPRY1 (**C**), and RECK (**D**) following TGF-β as well as pre-miR-21 and anti-miR-21 (“α-miR-21”) transfection, the latter performed during TGF-β incubation. Expression values (means+SD, n = 3) are displayed relative to the corresponding treatment controls (medium, pre-miR scramble, or anti-miR scramble) as visualized by the dotted lines intersecting “1”. Expression data were normalized against GAPDH and RPL13A, and statistical significance was tested by a two-tailed t-test. *P<0.05, **P<0.01 vs. the control. **E**, PDCD4 protein amounts (mean+SD, n = 3) were evaluated by Western blotting, and band intensities were quantified and standardized with corresponding bands from a Coomassie staining (total protein; not shown).

Next, we questioned how miR-21 exerts these substantial effects on fibroblast-committed EMT of EMCs. Not only has miR-21 been identified as a driver of cardiac disease, but also substantially implicated and characterized in cancer conditions, and from these studies a plethora of direct miR-21 targets have been validated (by e.g. luciferase reporter assays), especially PDCD4, SPRY1, and reversion-inducing-cysteine-rich with kazal motifs (RECK), which regulates biological processes inherent to EMT [44]. Testing these putative targets, we found that PDCD4 and SPRY1 were expressed in EMCs, with a nucleic and cytosolic localization, respectively, and that TGF-β treatment substantially reduced the levels of these proteins, concomitant to the up-regulation of α-SMA (Fig. 4A). RECK was not assessed on the protein level in EMCs, but following TGF-β incubation for 48****h the RECK mRNA expression was significantly reduced (*P* = 0.0217; Fig. 4D), along with PDCD4 (*P* = 0.0040; Fig. 4B) and SPRY1 (*P* = 0.0414; Fig. 4C) mRNA expression (Figure 4B). To evaluate if these target reductions were consequences of miR-21 targeting, we measured the mRNA expression of PDCD4, SPRY1, and RECK following EMC transfection with pre-miR-21 or anti-miR-21, the latter performed during TGF-β-induced EMT. Hence, we found that PDCD4 (Fig. 4B) and to a lesser extent SPRY1 (Fig. 4C) were significantly targeted by miR-21 in EMCs, while RECK (Fig. 4D) was not, and we thus focused on PDCD4 and SPRY1 in the remaining part of the study. We then confirmed that also the protein level of PDCD4 was significantly rescued by anti-miR-21 (1.66±0.11, *P* = 0.0086; Fig. 4E), while SPRY1 protein regulation was experimentally undetectable (results not shown).

### MiR-21 Promotes a Mesenchymal Phenotype in an Interplay with PDCD4 and SPRY1

We finally hypothesized that the observed effects of miR-21 on EMT biomarkers (Table 1) was a result of its direct down-regulation of PDCD4 and/or SPRY1. We therefore co-transfected TGF-β-stimulated EMCs with anti-miR-21 and either a control siRNA (α-CTL), a siRNA against PDCD4 (α-PDCD4), or a siRNA against SPRY1 (α-SPRY1). Hence, by analyzing biomarker read-outs by one-way ANOVAs, we could assess whether miR-21 regulated the EMT biomarkers through PDCD4 and/or SPRY1 in case the effect from inhibiting miR-21 by anti-miR-21 was lost when PDCD4 or SPRY1 was concurrently targeted by siRNAs.

**Figure 5:**
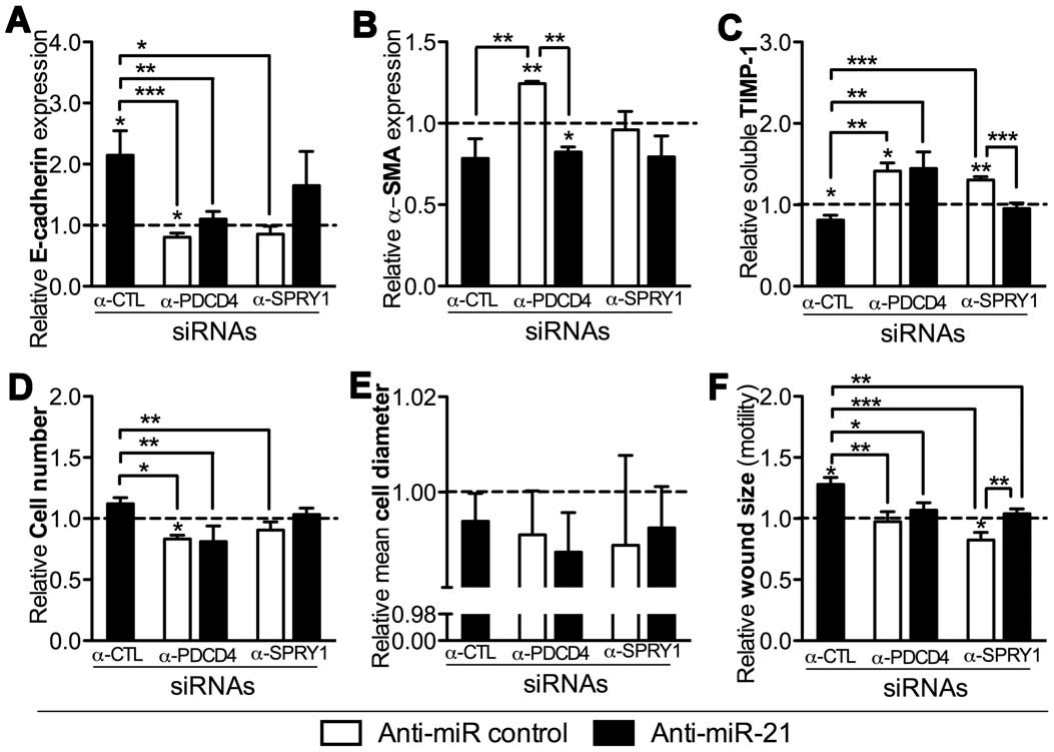
PDCD4 and SPRY1 regulate biomarkers of fibrogenic EMT in EMCs, partly via miR-21. TGF-β-incubated (20****h) EMCs were co-transfected with an anti-miR control or an anti-miR-21 along with siRNAs against PDCD4 or SPRY (or siRNA-control; “α-CTL”), and analyzed after 24****h for E-cadherin expression (**A**), α-SMA expression (**B**), TIMP-1 (**C**) in the culture medium (ELISA) as well as cell number (**D**), mean cell diameter (**E**), and cell motility (**F**) by the relative wound size closure in a scratch assay. qRT-PCR data were normalized against GAPDH and RPL13A. All data are presented as means+SD, n = 3 relative to the control treatment (α-CTL+anti-miR control). Statistical significance was tested by one-sample t-tests for each treatment and one-way ANOVAs followed by Tukey’s post test compared treatment effects within the PDCD4- and SPRY1-specific experiments. *P<0.05, **P<0.01, ***P<0.001.

**Figure 6:**
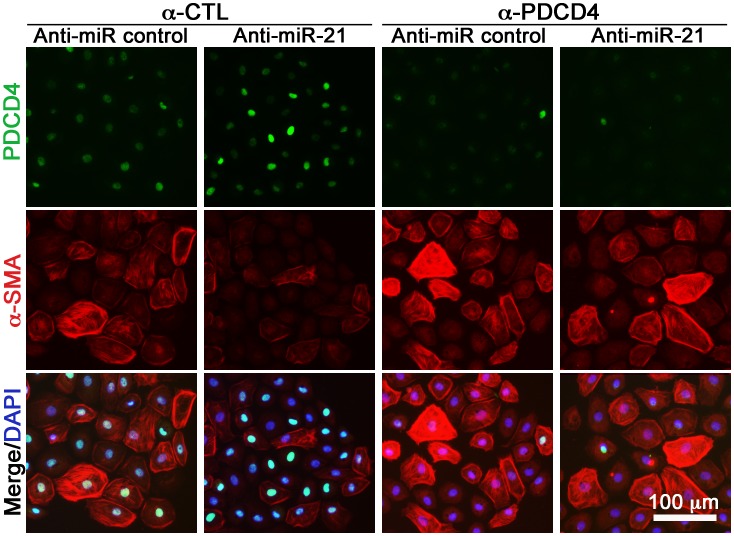
PDCD4 and α-SMA immunofluorescence of TGF-β-incubated EMCs. EMCs were pre-incubated for 20****h in TGF-β, then co-transfected with anti-miR-21 (or anti-miR control) and/or PDCD4-specific siRNA (or siRNA control; “α-CTL”), and stained after 24****h. Control for immunofluorescence is in Figure 4A.

**Figure 7:**
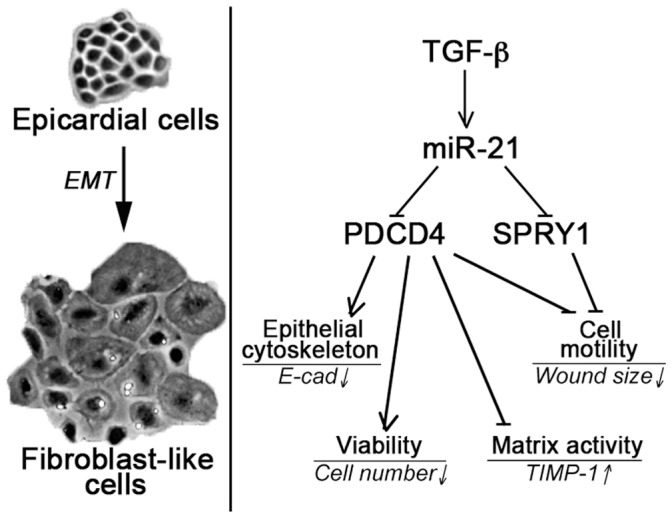
Concepts of cardiac fibrogenic EMT. Adult EMCs undergo EMT to generate fibroblast-like cells through up-regulation of miR-21, which in turn directly targets, and hence suppresses, PDCD4 and SPRY1. On the other hand, PDCD4 and SPRY1 can regulate various EMT biomarkers through miR-21-dependent mechanisms.

In accordance with Table 1, we again found that all EMT biomarkers were regulated by miR-21 (Figs. 5 and 6), except the mean cell diameter (Fig. 5E). E-cadherin expression (Fig. 5A), TIMP-1 (Fig. 5C), cell number (Fig. 5D), and cell motility (Fig. 5F) were regulated through PDCD4 via miR-21 since α-CTL+anti-miR-21 vs. α-PDCD4+α-miR-21 was significant different in these instances. The investigated EMT biomarkers were less regulated by SPRY1, implicating only significant miR-21-dependent targeting in case of cell motility (α-CTL+anti-miR-21 vs. α-PDCD4+α-miR-21, *P*<0.01; Fig. 5F and Fig. S8). These data thus suggest that miR-21 regulates fibrogenic cardiac EMT through direct targeting of PDCD4 and SPRY1 (Fig. 7), but that other miR-21 specific targets must also be involved to account for the measured phenotypic effects of anti-miR-21 (Table 1).

## Discussion

In the present study, we support other reports on the existence of adult fibrogenic EMT of the EMCs lining the epicardium [4], [5], [6], [7], [8] and propose a model of how fibrogenic EMT of EMCs is controlled at the level of miRNAs (Fig. 7). We implicate TGF-β as a far more potent EMT-inducer in EMCs than TNF-α and especially IL-1β, and detected substantial differentiation into myofibroblastic cells for TGF-β incubation, as measured by various markers, including development of stress fibers of α-SMA (Fig. 2C). Despite this differential potency in inducing EMT, miR-21 was the most up-regulated miRNA induced by all three stimuli.

Notably, IL-1β and TNF-α induced miR-21 at equal levels (Fig. 3B), but TNF-α was far more potent in inducing EMT, which is likely an outcome of differential induction of the molecular pool unrelated to miR-21 effects. As such, the more diverse TNF-α signaling, including caspase 8-mediated death signaling [45], might promote EMT more compared to the classic NF-kB/MAPK pathways of IL-1β [46], or that IL-1β also induces factors that act to hamper EMT, while TNF-α may not.

Importantly, miR-21 manipulations showed substantial impact on EMC phenotype, since overexpression of miR-21 in EMCs markedly promoted biomarkers of fibrogenic EMT, while fibrogenic EMT was substantially inhibited when a miR-21 antagonist was present during EMT (Table 1). We observed more impact from the miR-21 antagonist than the miR-21 mimic. This is likely because anti-miR-21 transfection was performed 20****h after EMCs had undergone TGF-β treatment and readouts assessed after another 24****h of incubation in TGF-β, while Pre-miR-21 transfection was performed on resting EMCs and the phenotype was evaluated after 48****h.

Our results indentify miR-21 as a key regulator of fibrogenic EMT of EMCs, which is in parallel to an increasing number of reports documenting high significance of miR-21 in cardiac diseases. As reported by several independent groups, analyses on both patient samples [17], [18], [19], [20] along with animal injury models [17], [23], [41], [42], [43], [47], [48], miR-21 is implicated in cardiac hypertrophy, proliferative vascular disease and ischemic heart disease. We furthermore show that miR-21 is up-regulated in a time-dependent manner during TAC (Fig. 3C) and LAD (Fig. 3D), and not only in later strict fibrosis-associated phases, but that miR-21 up-regulation is already substantial at very early time-points dominated by inflammatory phases, characterized by e.g. high levels of pro-inflammatory molecules such IL-1β [46] and TNF-α [45]. However, recent contradictory results on the involvement of miR-21 in cardiac disease imply that more studies are needed to elucidate miR-21′s role in the heart [49], [50].

Nevertheless, miR-21 has been associated with cell survival and hypertrophy in cardiomyocytes [23], [43], [47], whereas in vascular smooth muscle cells and fibroblasts, miR-21 promotes cellular activity in the direction of remodeling, which can be reduced or even reversed by miR-21 antagonists [22], [23], [41], [51], [52], supporting our results. Additionally, we show that the miR-21-mediated PDCD4- and SPRY1-targeting promotes a number of mesenchymal, fibrogenic markers in EMCs. We therefore propose that miR-21 mediates cardiac remodeling also through EMT of EMCs, partly through a PDCD4- and SPRY1-dependent mechanism (Fig. 7). As such, miR-21 may skew the EMC progenitor potential towards remodeling, rather than regeneration.

At the molecular level, several miR-21 targets have been experimentally validated in several biological systems. In cardiac disease, functional assessment of miR-21 implicates PDCD4 [41], [48], [52], [53], SPRY1 [22] or SPRY2 [54], Phosphatase and tensin homolog [23], [29], [55], and Fas ligand [55] as the major targets of miR-21. Our data solidly support the involvement of PDCD4 and SPRY1 in miR-21-regulated fibrogenic processes in the heart. However, the phenotypic measurements also suggest that other targets of miR-21 are involved in cardiac fibrogenic EMT, since e.g. α-SMA was regulated through a miR-21-dependent mechanism (Table 1), which could not be proven to occur through PDCD4 or SPRY1 (Figs. 5B and 6). This supports the notion that a single miRNA may regulate several target mRNAs [56] and furthermore supports that miR-21 is a pleiotropic acting miRNA within biological systems such as cancers and cardiovascular diseases [57]. In this study, we focused on miR-21 targets relating downstream to the MAPK/NF-κB pathways as all three tested EMT inducers can signal through those, but in relation to TGF-β-induced EMT it would be relevant to further explore miR-21 targets relating the TGF-β pathway to account for the missing functional effect from miR-21 at the level of downstream targets (Fig. 5). However, Smad7, which have been shown to be a miR-21 target in pulmonary fibrosis [58], does not appear to be implicated in our culture system, since we registered no significant regulation of Smad7 expression during EMT (Fig. S9).

Our miRNA array data show many differentially regulated miRNAs besides miR-21 (Fig. 3 and Table S2). However, the effects from miR-21 account for up to 50% or even more on the investigated fibrogenic EMT biomarkers (Table 1), substantiating that miR-21 is not only the most regulated miRNA, but also a functionally outmost important miRNA during cardiac fibrogenic EMT. Similar to our results, two reports on TGF-β-induced EMT in human keratinocytes [24] and a colon carcinoma cell line [27] both identified miR-21 as the major up-regulated miRNA during EMT, and a recent study on TGF-β-induced cardiac EndMT showed that miR-21 promoted EndMT through targeting of the PTEN/Akt pathway [29]. In addition, miR-21 targeting of PDCD4 and RECK has been identified as a driver of branching morphogenesis of the epithelium in the submandibular gland [59]. However, other *in vitro* studies of TGF-β-induced EMT have identified miR-155, by RhoA targeting [60], and especially the miR-200 family in tumor metastasis, by targeting E-cadherin transcriptional repressors ZEB1/ZEB2 [25], [26], [28], as EMT-promoting miRNAs. Owing to the different nature of the epithelial cell lines used in those studies compared to the mesothelial cardiac origin of the EMCs in our study, the miRNA fingerprint along with the differential miRNA expression during EMT is expected to differ substantially. Furthermore, since rat-derived EMCs were used, and miR-155 is not annotated in the rat (miRBase 10.0–15.0), we could not assess miR-155 in our analyses.

In summary, miR-21 promotes robust fibrogenic EMT of EMCs, in part by directly targeting PDCD4 and SPRY1. Due to the multipotency of EMCs, our results implicate miR-21 as a relevant target to reduce the differentiation of EMCs into fibroblast-like cells, which may favor commitment into the vasculogenic/angiogenic or even the myogenic lineage.

## Supporting Information

Figure S1 **Validation of EMC culture purity.** **A**, The EMC culture in passage 3 displayed neither CD45 nor, **B**, Troponin T expression, as evaluated by immunofluorescence. **C**, Staining for α-smooth muscle actin (α-SMA) was only marginally positive and showed no stress fiber formation. For all stainings, the signal was adjusted to the specific isotype controls. The specificity of antibodies has previously been tested positive (27) within the same range of exposure. **D**, The CD31 surface marker expression of EMCs (upper panel; means±SD) in passage 3 was tested by flow cytometry and was negative compared to the stromal vascular fraction fraction (lower panel), showing that the cells did not have an endothelial commitment. Green and purple histograms illustrate analysis for the isotype control and the specific antibody, respectively.
(TIF)

Figure S2 **Enrichment of transcriptional EMT biomarkers in EMC cultures.** The expression of classic EMT inducers, Twist-1, Snail, and Slug, was tested for enrichment in adult rat EMCs (n = 3) relative to an embryonic (Em.) and an adult (Ad.) rat heart. Expression (means+SD) was measured by qRT-PCR and presented relative to GAPDH and RPL13A.
(TIF)

Figure S3 **The phenotype changes differentially depending on EMT-inducing stimuli.** The morphological effects were measured by Coulter measurements (**A**, **B)**, and a standard MTT assay (**C**). **D**, TIMP-1 in supernatants was measured by ELISA to assess the matrix activity. Data (means+SD, n = 3) are presented relative to the values at the time when stimulants were added, and statistical significance was tested by one-way ANOVA at the given time point and treatment effects were assessed by Tukey’s post test. *P<0.05, **P<0.01, ***P<0.001 vs. the control (CTL).
(TIF)

Figure S4 **Evaluation of pre- and anti-miR transfection efficiency in EMCs.** Three EMC cultures were pooled and transfected with the indicated concentrations of, **A**, a FAM-conjugated pre-miR scramble control or, **B**, a FAM-conjugated anti-miR scramble control. The FAM signal was computed by flow cytometry immediately after transfection. The acquired FAM-signal is represented as both the percentage of positive events (left y-axis/black bars) or as the geometric mean intensity (right y-axis/white bars). Lipofectamine was required for transfection as indicated by the diminished signal when Lipofectamine 2000 was omitted from the probe preparation. In both the computation of pre- and anti-miR transfection efficiency, cell necrosis was tested by Propidium Iodide staining, showing only <4% positive events by flow cytometry (results not shown).
(TIF)

Figure S5 **Motility of EMCs measured by the relative wound sizes at 0 h versus 21 h after scratches were induced.** **A**, The effect of TGF-β on wound closure in EMC cultures is illustrated from one representative experiment. **B**, Representative phase pictures for the effect of pre-miR-21 transfection of EMCs on wound size. **C**, Also representative phase pictures illustrating wound closure, in this instance for transfection of EMC with anti-miR-21. In contrast to pre-miR-21 transfection, EMCs had initially been pre-incubated in TGF-β and then transfected for 4****h with anti-miR-21. Subsequently, the scratch was induced and cells incubated for another 21****h in TGF-β. The wound distance was measured at the two time points in biological triplicates as a mean of the wound distance at three pre-defined spots on the plate. The differential wound size was quantified relative to the control, as presented in Table 1.
(TIF)

Figure S6 **Efficacy of siRNAs.** Selection of the most efficient siRNA variant for, **A**, PDCD4 or, **B**, SPRY1 mRNA knockdown. For each gene product, three variants of Silencer Select siRNAs were evaluated by performing expression analyses 48****h after transfection. Expression levels (means+SD, n = 3) of PDCD4 and SPRY1 were normalized with those of GAPDH and RPL13A, and presented relatively to the siRNA control. Statistical significance was tested by a two-tailed t-test for each variant.
(TIF)

Figure S7 **Verification of transfection efficiency for combined anti-miR and siRNA transfections. A**, A pool of three EMC cultures were transfected with varying concentrations of a FAM-conjugated siRNA control in the presence of 20 nmol/L anti-miR control or, **B**, with varying concentrations of a FAM-conjucated anti-miR control in the presence of 20 nmol/L siRNA control. The FAM-signal was aquired by flow cytometry immediately after transfection and represented as both the percentage of positive events (left y-axis/black bars) or as the geometric mean intensity (right y-axis/white bars). Cell necrosis was tested by Propidium Iodide staining, showing only <2% positive events by flow cytometry (results not shown).
(TIF)

Figure S8 **Effect of transfections on motility of EMCs.** The relative wound sizes were assessed at 0****h (**A**) versus 21****h (**B**) after scratches were induced. The wound distance was measured at the two time points in biological triplicates as a mean of the wound distance at three pre-defined spots on the plate. Phase pictures are representative for one of three biological experiments and represent EMCs transfected with the indicated combinations of anti-miR and siRNAs, directed against PDCD4 or SPRY1, during TGF-β-induced EMT.
(TIF)

Figure S9 **Smad7 expression during fibrogenic EMT of EMCs.** Non-confluent EMC cultures were stimulated with IL-1β, TNF-α, or TGF-β, and Smad7 expression was measured after 48 and 96****h by qRT-PCR. Data (means+SD, n = 3) were normalized against GAPDH and RPL13A, and statistical significance was tested by one-way ANOVA, revelaing no significant difference between treatments and the control (CTL) at the indicated time-points.
(TIF)

Table S1 **Primer sequences.**
(DOC)

Table S2 **The 50 most statistically significant regulated (** ***P*** **<0.05) miRNAs in cardiac EMT.** The differential miRNA expression was measured in EPDC cultures incubated for 48****h with 10 ng/ml IL-1β, TNF-α or TGF-β versus the control treatment.
(DOC)
