# Supplementary figures and images for: miR-21 Promotes Fibrogenic Epithelial-to-Mesenchymal Transition of Epicardial Mesothelial Cells Involving Programmed Cell Death 4 and Sprouty-1
Source: PLoS One. 2013 Feb 18;8(2):e56280. doi: 10.1371/journal.pone.0056280 (PMC3575372; doi:10.1371/journal.pone.0056280)

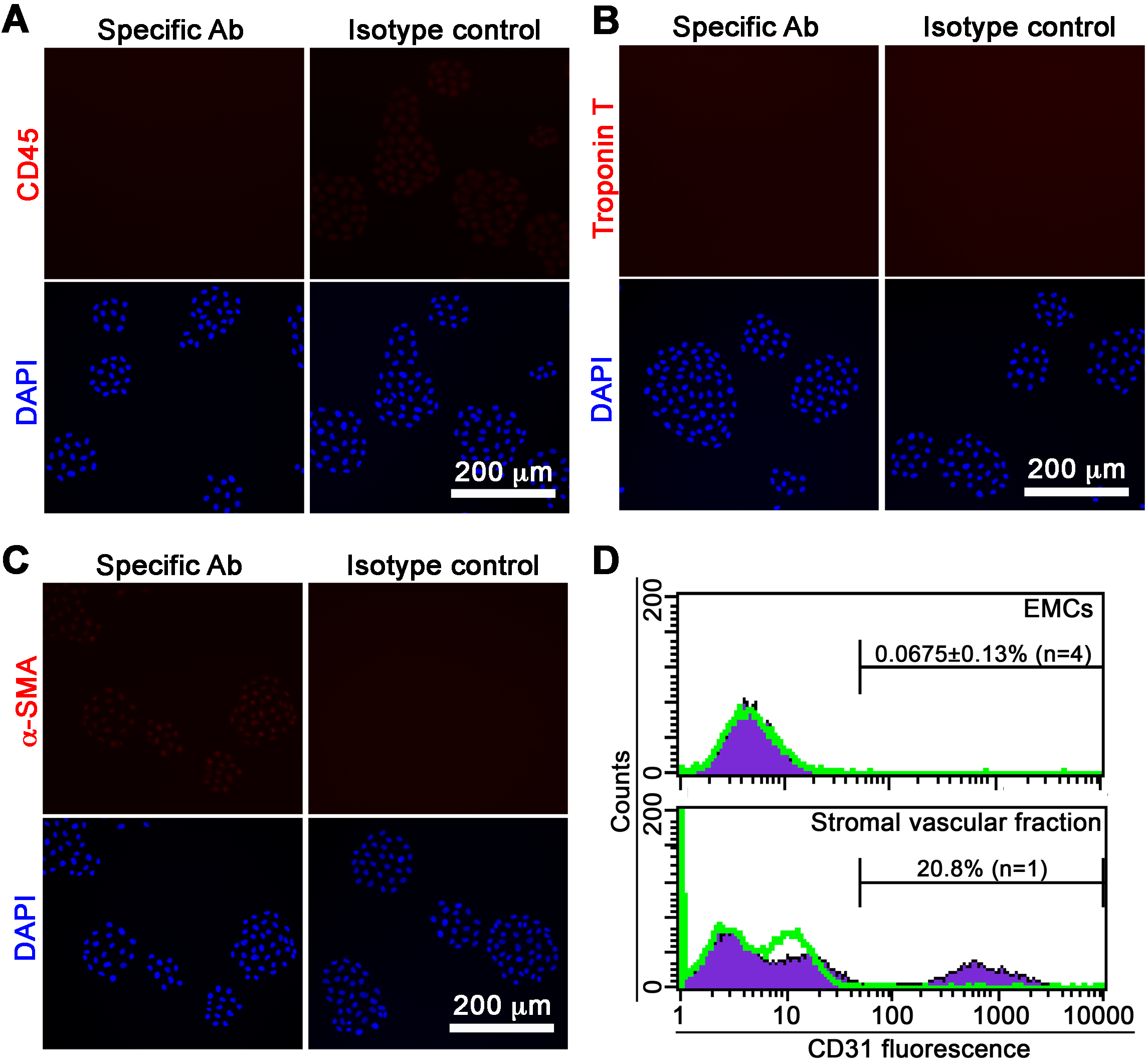

Supplement: Figure S1 — Validation of EMC culture purity. A, The EMC culture in passage 3 displayed neither CD45 nor, B, Troponin T expression, as evaluated by immunofluorescence. C, Staining for α-smooth muscle actin (α-SMA) was only marginally positive and showed no stress fiber formation. For all stainings, the signal was adjusted to the specific isotype controls. The specificity of antibodies has previously been tested positive (27) within the same range of exposure. D, The CD31 surface marker expression of EMCs (upper panel; means±SD) in passage 3 was tested by flow cytometry and was negative compared to the stromal vascular fraction fraction (lower panel), showing that the cells did not have an endothelial commitment. Green and purple histograms illustrate analysis for the isotype control and the specific antibody, respectively. (TIF) [file pone.0056280.s001.tif]

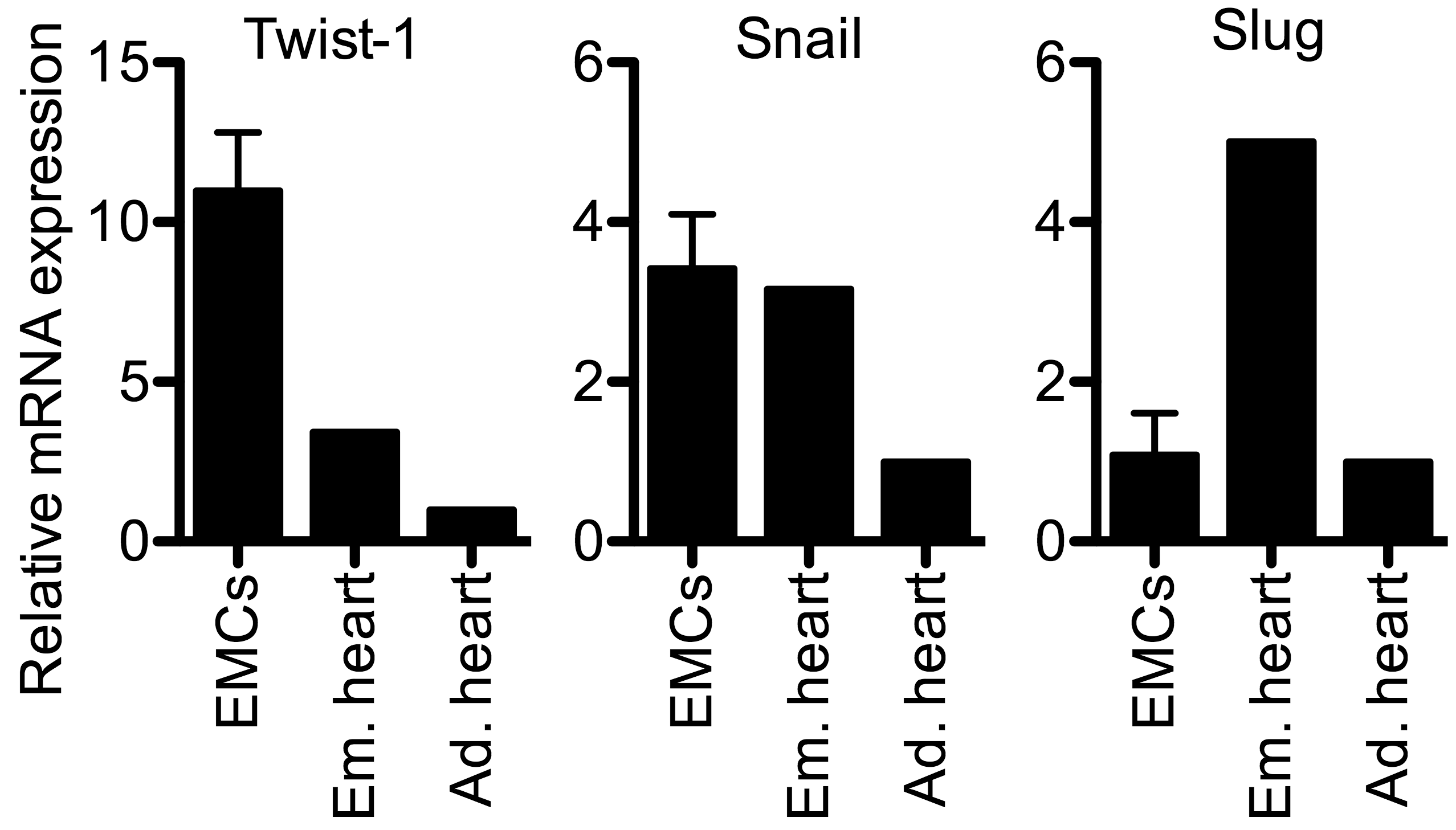

Supplement: Figure S2 — Enrichment of transcriptional EMT biomarkers in EMC cultures. The expression of classic EMT inducers, Twist-1, Snail, and Slug, was tested for enrichment in adult rat EMCs (n = 3) relative to an embryonic (Em.) and an adult (Ad.) rat heart. Expression (means+SD) was measured by qRT-PCR and presented relative to GAPDH and RPL13A. (TIF) [file pone.0056280.s002.tif]

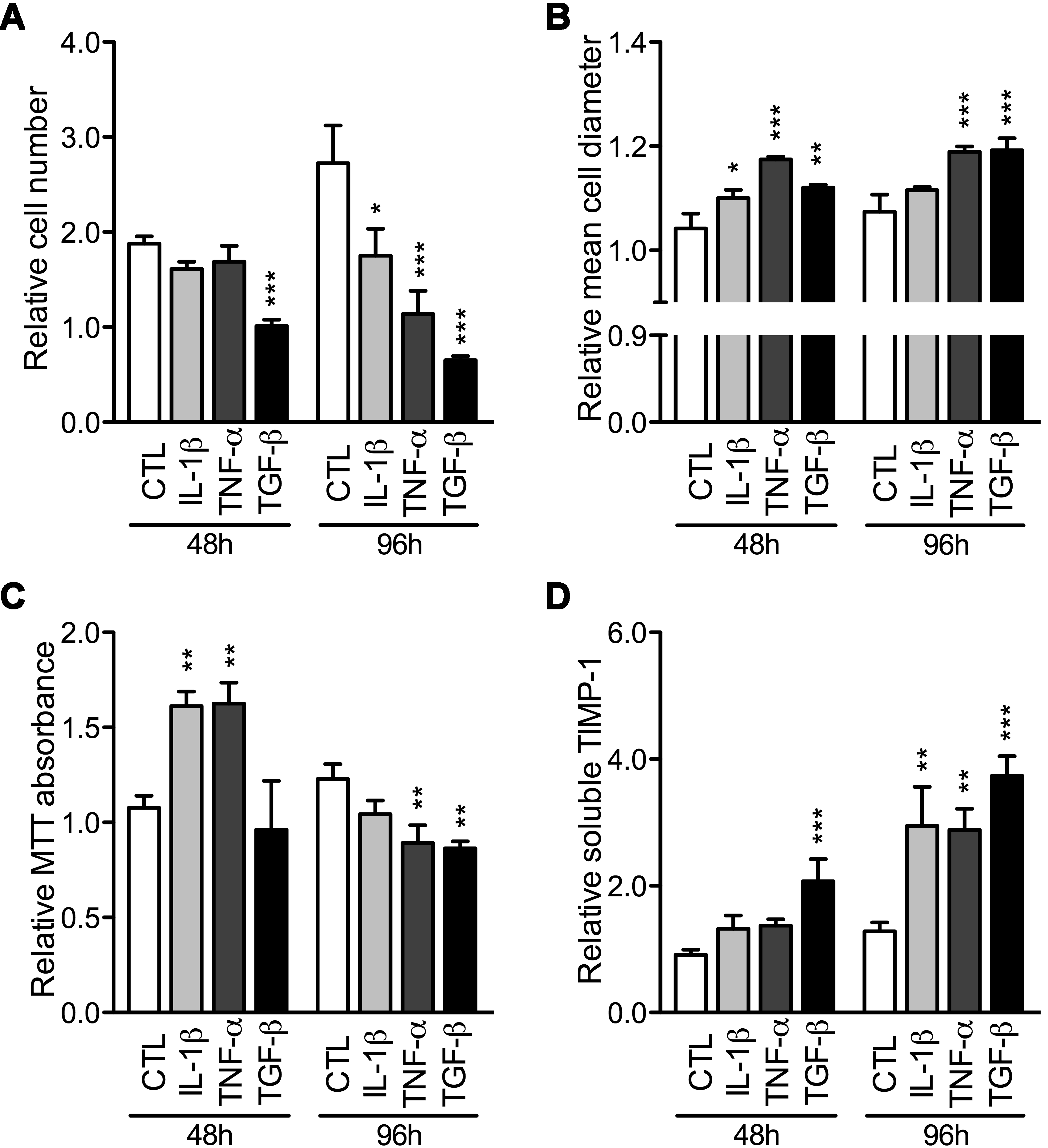

Supplement: Figure S3 — The phenotype changes differentially depending on EMT-inducing stimuli. The morphological effects were measured by Coulter measurements (A, B), and a standard MTT assay (C). D, TIMP-1 in supernatants was measured by ELISA to assess the matrix activity. Data (means+SD, n = 3) are presented relative to the values at the time when stimulants were added, and statistical significance was tested by one-way ANOVA at the given time point and treatment effects were assessed by Tukey’s post test. *P<0.05, **P<0.01, ***P<0.001 vs. the control (CTL). (TIF) [file pone.0056280.s003.tif]

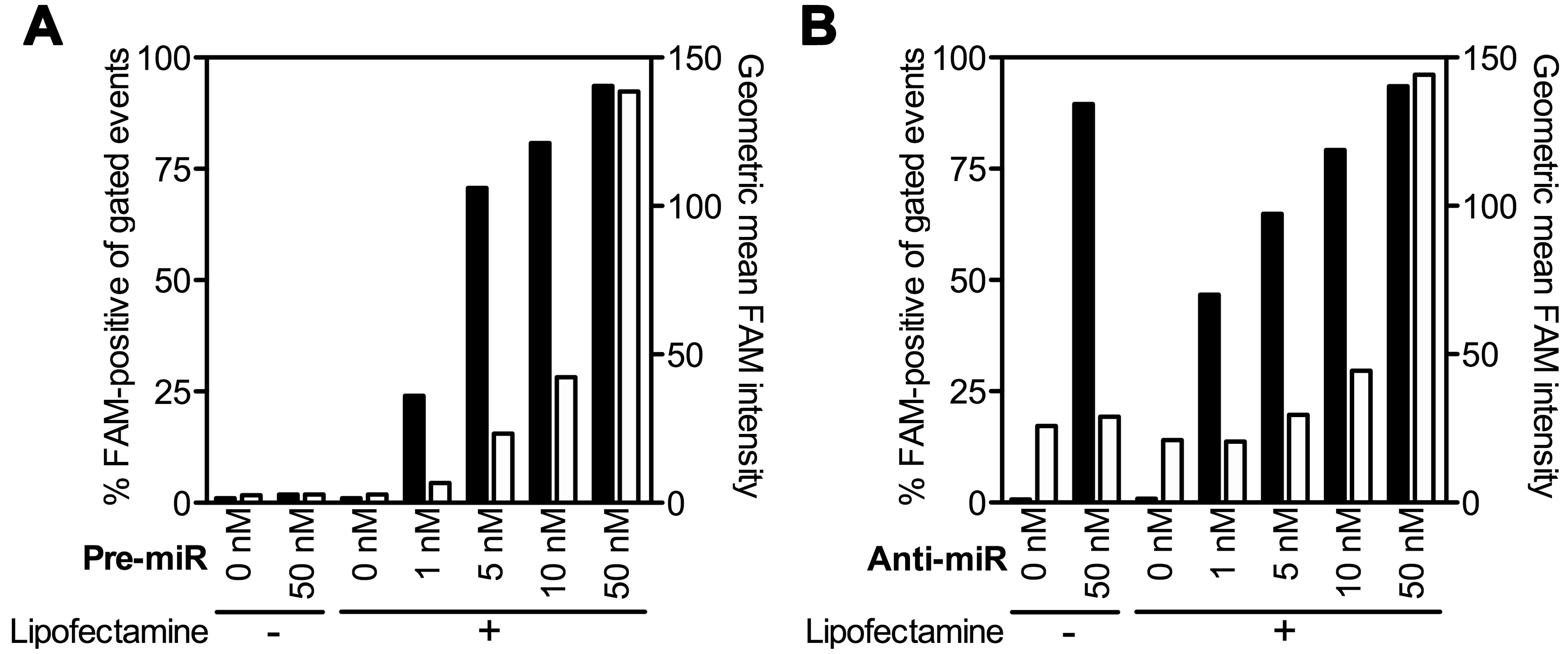

Supplement: Figure S4 — Evaluation of pre- and anti-miR transfection efficiency in EMCs. Three EMC cultures were pooled and transfected with the indicated concentrations of, A, a FAM-conjugated pre-miR scramble control or, B, a FAM-conjugated anti-miR scramble control. The FAM signal was computed by flow cytometry immediately after transfection. The acquired FAM-signal is represented as both the percentage of positive events (left y-axis/black bars) or as the geometric mean intensity (right y-axis/white bars). Lipofectamine was required for transfection as indicated by the diminished signal when Lipofectamine 2000 was omitted from the probe preparation. In both the computation of pre- and anti-miR transfection efficiency, cell necrosis was tested by Propidium Iodide staining, showing only <4% positive events by flow cytometry (results not shown). (TIF) [file pone.0056280.s004.tif]

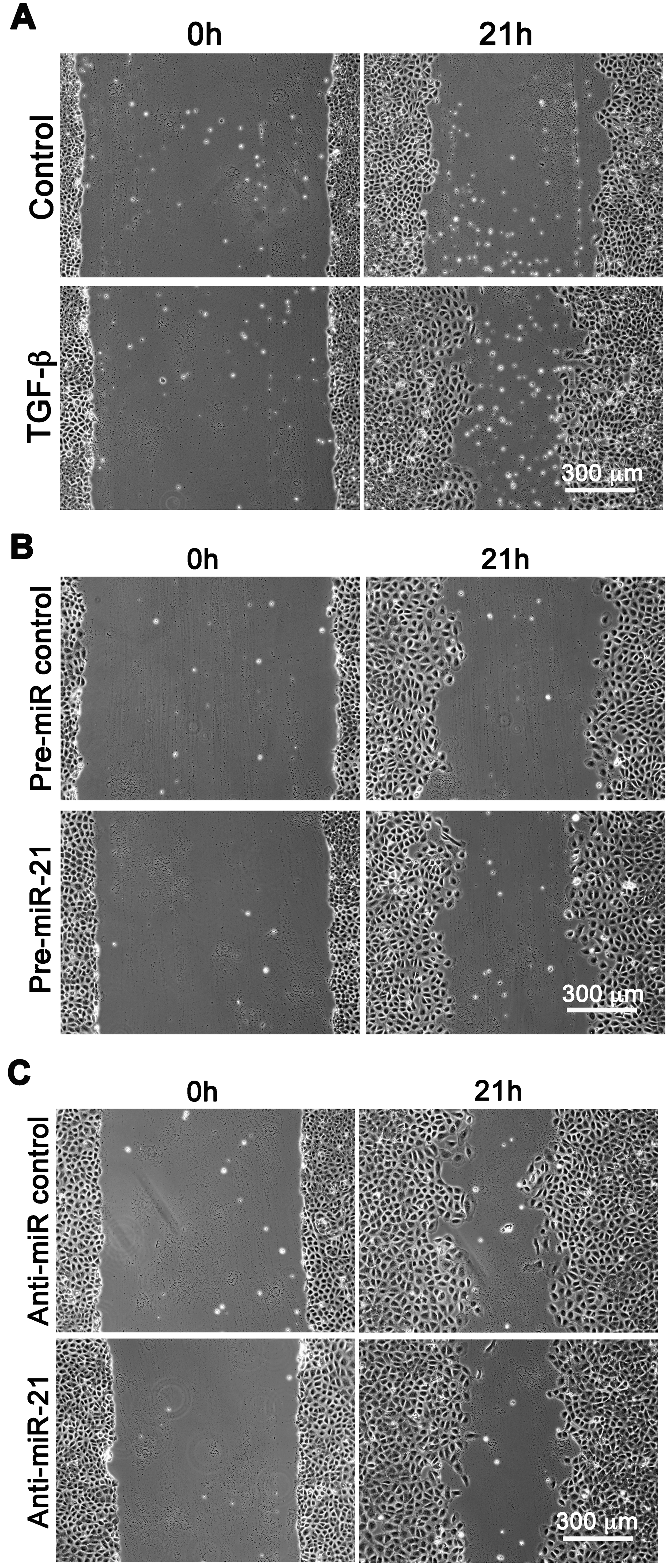

Supplement: Figure S5 — Motility of EMCs measured by the relative wound sizes at 0 h versus 21 h after scratches were induced. A, The effect of TGF-β on wound closure in EMC cultures is illustrated from one representative experiment. B, Representative phase pictures for the effect of pre-miR-21 transfection of EMCs on wound size. C, Also representative phase pictures illustrating wound closure, in this instance for transfection of EMC with anti-miR-21. In contrast to pre-miR-21 transfection, EMCs had initially been pre-incubated in TGF-β and then transfected for 4h with anti-miR-21. Subsequently, the scratch was induced and cells incubated for another 21h in TGF-β. The wound distance was measured at the two time points in biological triplicates as a mean of the wound distance at three pre-defined spots on the plate. The differential wound size was quantified relative to the control, as presented in Table 1. (TIF) [file pone.0056280.s005.tif]

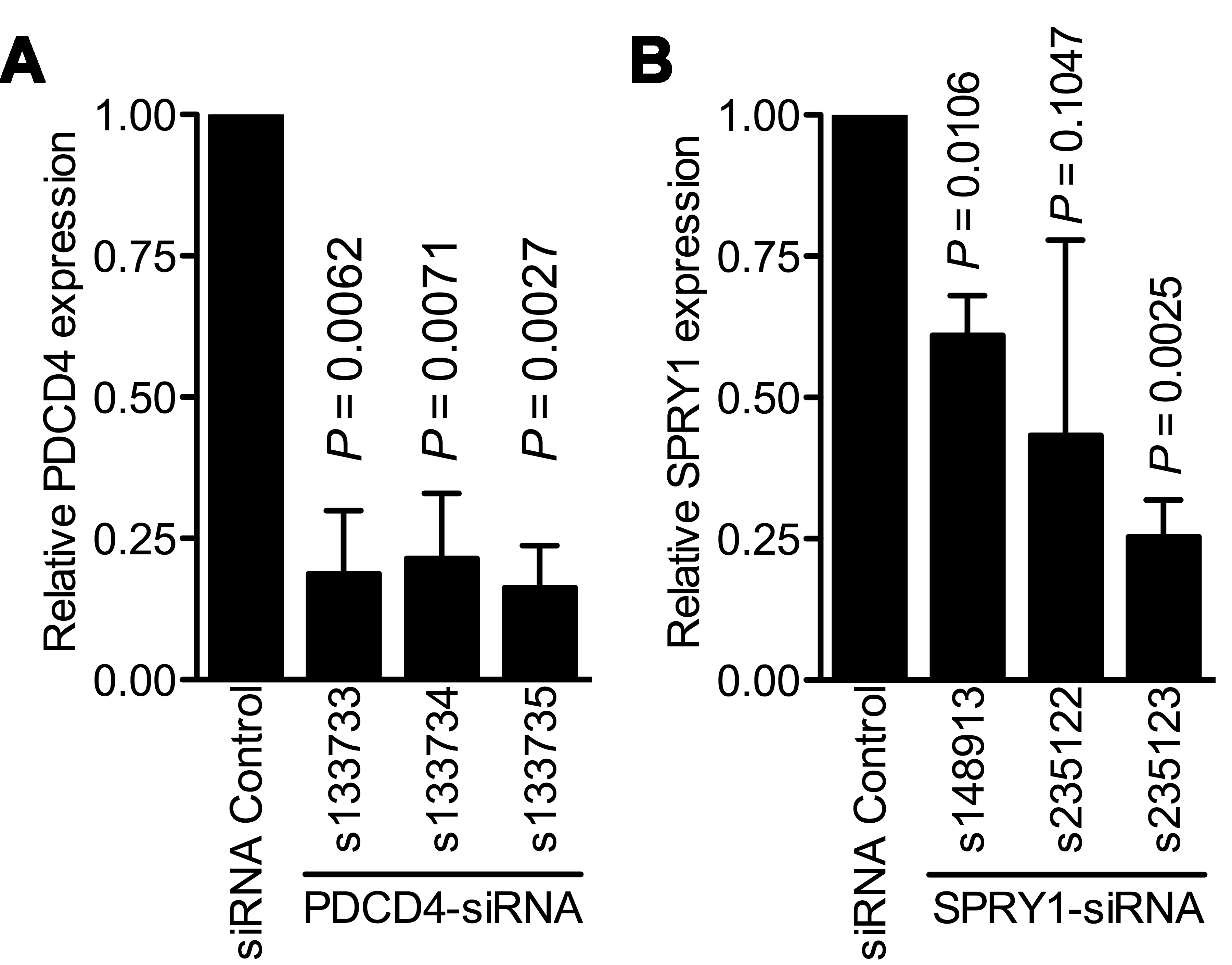

Supplement: Figure S6 — Efficacy of siRNAs. Selection of the most efficient siRNA variant for, A, PDCD4 or, B, SPRY1 mRNA knockdown. For each gene product, three variants of Silencer Select siRNAs were evaluated by performing expression analyses 48h after transfection. Expression levels (means+SD, n = 3) of PDCD4 and SPRY1 were normalized with those of GAPDH and RPL13A, and presented relatively to the siRNA control. Statistical significance was tested by a two-tailed t-test for each variant. (TIF) [file pone.0056280.s006.tif]

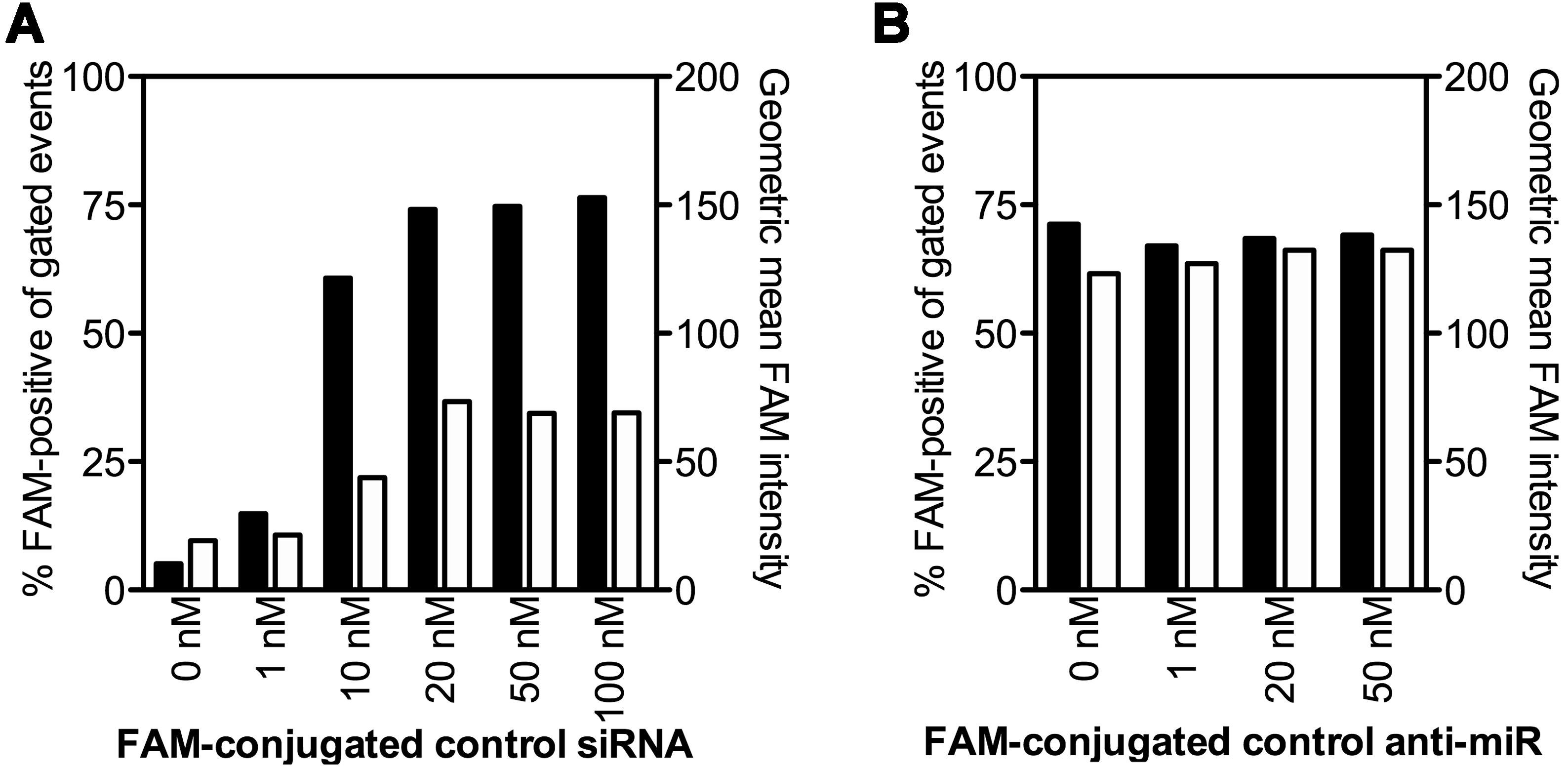

Supplement: Figure S7 — Verification of transfection efficiency for combined anti-miR and siRNA transfections. A, A pool of three EMC cultures were transfected with varying concentrations of a FAM-conjugated siRNA control in the presence of 20 nmol/L anti-miR control or, B, with varying concentrations of a FAM-conjucated anti-miR control in the presence of 20 nmol/L siRNA control. The FAM-signal was aquired by flow cytometry immediately after transfection and represented as both the percentage of positive events (left y-axis/black bars) or as the geometric mean intensity (right y-axis/white bars). Cell necrosis was tested by Propidium Iodide staining, showing only <2% positive events by flow cytometry (results not shown). (TIF) [file pone.0056280.s007.tif]

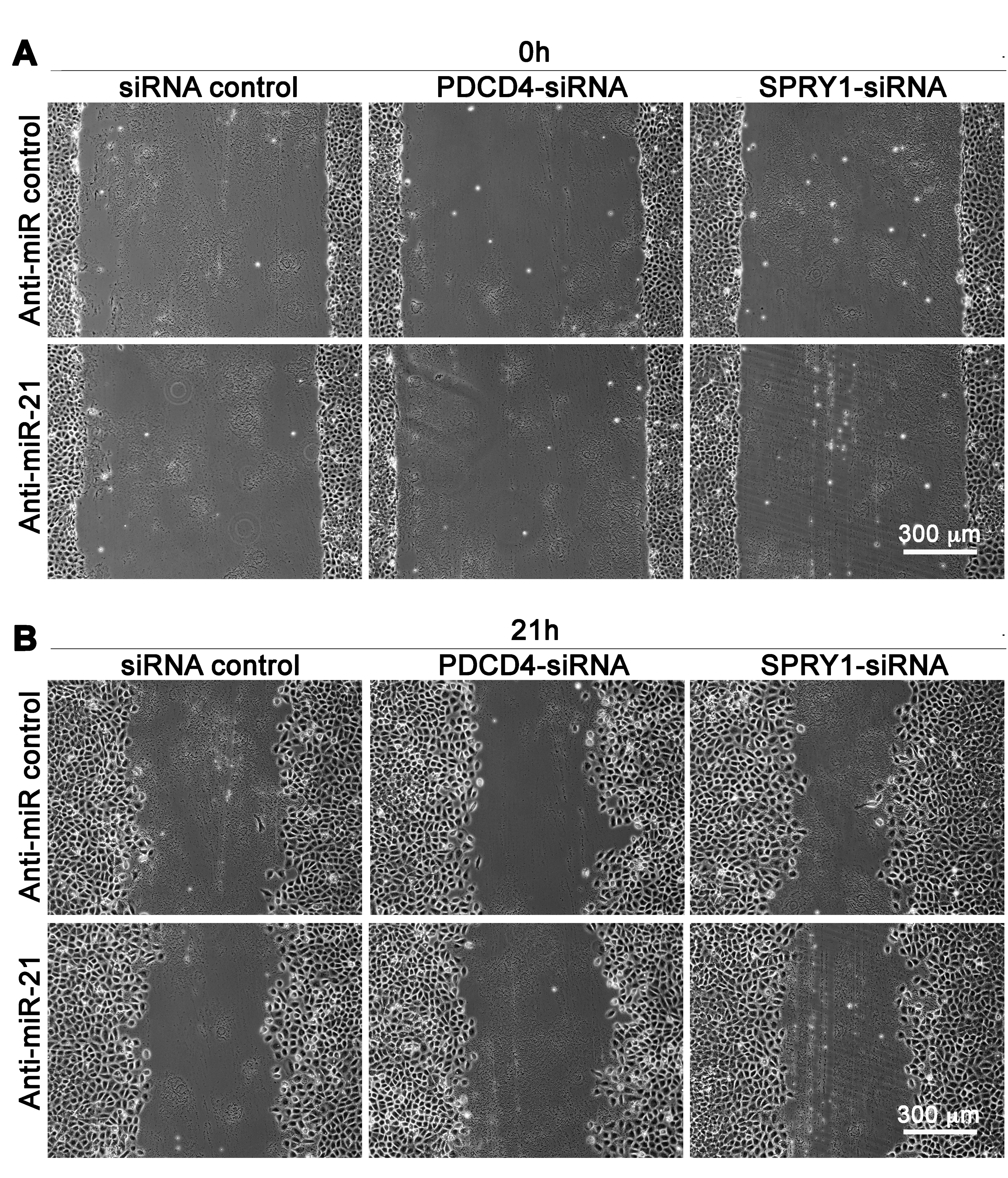

Supplement: Figure S8 — Effect of transfections on motility of EMCs. The relative wound sizes were assessed at 0h (A) versus 21h (B) after scratches were induced. The wound distance was measured at the two time points in biological triplicates as a mean of the wound distance at three pre-defined spots on the plate. Phase pictures are representative for one of three biological experiments and represent EMCs transfected with the indicated combinations of anti-miR and siRNAs, directed against PDCD4 or SPRY1, during TGF-β-induced EMT. (TIF) [file pone.0056280.s008.tif]

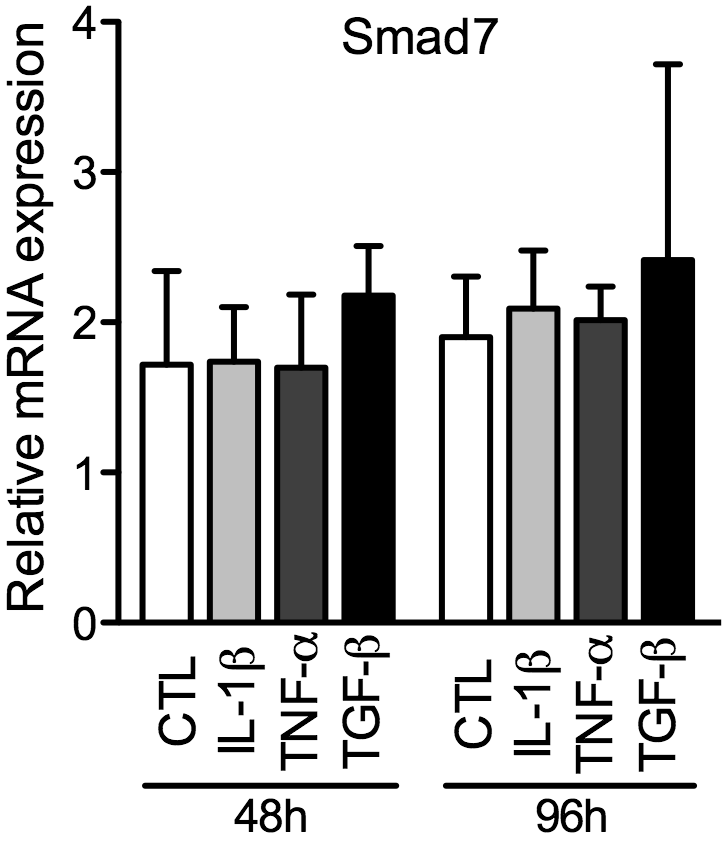

Supplement: Figure S9 — Smad7 expression during fibrogenic EMT of EMCs. Non-confluent EMC cultures were stimulated with IL-1β, TNF-α, or TGF-β, and Smad7 expression was measured after 48 and 96h by qRT-PCR. Data (means+SD, n = 3) were normalized against GAPDH and RPL13A, and statistical significance was tested by one-way ANOVA, revelaing no significant difference between treatments and the control (CTL) at the indicated time-points. (TIF) [file pone.0056280.s009.tif]
