# Supplementary material for: miR-21 Promotes Fibrogenic Epithelial-to-Mesenchymal Transition of Epicardial Mesothelial Cells Involving Programmed Cell Death 4 and Sprouty-1
Source: PLoS One. 2013 Feb 18;8(2):e56280. doi: 10.1371/journal.pone.0056280 (PMC3575372; doi:10.1371/journal.pone.0056280)
Supplement: Table S1 — Primer sequences. (DOC) [file pone.0056280.s010.doc]

| **Gene** |  | **Forward (5’-3’)** | **Reverse (5’-3’)** |  | **Amplicon size** |
| --- | --- | --- | --- | --- | --- |
|  |  |  |  |  |  |
| E-cadherin |  | TCCATGCCTGGGACTCCAGTTAC | GAGCAGCTCTGGGTTGGATTCAG |  | 122 bp |
| Islet1 |  | AAGGACAAGAAACGCAGCAT | GGCTGGTAACTTTGCACCTC |  | 164 bp |
| Nkx2.5 |  | CAAGTGCTCTCCTGCTTTCC | GACAGGTACCGCTGTTGCTT |  | 253 bp |
| Tbx18 |  | CACCGAGGCCGACGAAGACC | CGTCCTCACAGCTGCCCGC |  | 128 bp |
| Raldh2 |  | TCCCGCACTGTGCTGTGGTA | GGGTGGAAAGCCAGCCTCCTTG |  | 106 bp |
| Twist |  | TCGTACGAGGAGCTGCAGACACA | CGGCAAATGCCTCGTTCAGCG |  | 85 bp |
| Snail |  | TCCAAACCCACTCGGATGTGAAGA | TTGGTGCTTGTGGAGCAAGGACAT |  | 86 bp |
| Slug |  | CACATTCGAACCCACACATTGCCT | TGTGCCCTCAGGTTTGATCTGTCT |  | 164 bp |
| α-smooth muscle actin |  | CAATGGCTCCGGGCTCTGTAAG | CGTCCCCCACATAGCTGTCCTT |  | 137 bp |
| PDCD4 |  | TCCAGGCCAGGGCTGCTTTG | TGACAGGCTGCTGCCCTCCT |  | 108 bp |
| SPRY1 |  | TCCCCGCACACAGCTCTGGA | TGTCCAAGCGCCCCCTCAGT |  | 149 bp |
| RECK |  | ACGTGTGACCAGGCCCGAGA | TGCAGAAAGGCTGGCACGGG |  | 121 bp |
| GAPDH |  | GTCGGTGTGAACGGATTTGGC | TGAAGGGGTCGTTGATGGCA |  | 97 bp |
| β-actin |  | TCTACAATGAGCTGCGTGTGGC | TGGGGTGTTGAAGGTCTCAAACA |  | 124 bp |
| RPL13A |  | GAAAGCGGATGAACACCAACCC | GGGATCCCATCCAACACCTTGA |  | 151 bp |
|  |  |  |  |  |  |
