# Supplementary material for: miR-21 Promotes Fibrogenic Epithelial-to-Mesenchymal Transition of Epicardial Mesothelial Cells Involving Programmed Cell Death 4 and Sprouty-1
Source: PLoS One. 2013 Feb 18;8(2):e56280. doi: 10.1371/journal.pone.0056280 (PMC3575372; doi:10.1371/journal.pone.0056280)
Supplement: Table S2 — The 50 most statistically significant regulated ( P <0.05) miRNAs in cardiac EMT. The differential miRNA expression was measured in EPDC cultures incubated for 48h with 10 ng/ml IL-1β, TNF-α or TGF-β versus the control treatment. (DOC) [file pone.0056280.s011.doc]

| **IL-1β treatment** | | | **TNF-α treatment** | | | **TGF-β treatment** | | |
| --- | --- | --- | --- | --- | --- | --- | --- | --- |
| miRNA | Fold Change | P- value | miRNA | Fold Change | P- value | miRNA | Fold Change | P- value |
| miR-21 | +2.22 | 0.0001 | miR-21 | +2.73 | 0.0000 | miR-21 | +3.15 | 0.0000 |
| miR-130a* | -2.01 | 0.0023 | miR-31 | +1.99 | 0.0000 | miR-371-5p | +2.29 | 0.0006 |
| miR-146b-5p | +2.00 | 0.0044 | miR-105 | +1.94 | 0.0002 | miR-933 | +2.23 | 0.0004 |
| miR-186* | -2.00 | 0.0063 | miR-181c | -1.85 | 0.0006 | miR-23a | +2.11 | 0.0061 |
| miR-105 | +1.89 | 0.0004 | miR-19b-1* | -1.81 | 0.0073 | miR-105 | +2.11 | 0.0001 |
| miR-876-3p | +1.89 | 0.0069 | miR-203* | +1.78 | 0.0065 | miR-888 | -2.11 | 0.0020 |
| miR-671-5p | -1.88 | 0.0082 | miR-199b-5p | -1.76 | 0.0057 | miR-668 | +2.09 | 0.0006 |
| miR-619 | -1.86 | 0.0276 | miR-523 | -1.76 | 0.0199 | let-7e | +2.04 | 0.0018 |
| miR-675 | -1.86 | 0.0202 | miR-608 | -1.76 | 0.0101 | miR-207 | +2.02 | 0.0003 |
| miR-146a | +1.85 | 0.0108 | miR-130a* | -1.76 | 0.0102 | miR-22 | +2.01 | 0.0047 |
| miR-941 | -1.82 | 0.0069 | miR-558 | -1.73 | 0.0056 | miR-879 | -2.00 | 0.0017 |
| miR-431* | -1.79 | 0.0271 | miR-93 | -1.72 | 0.0002 | miR-471 | +1.94 | 0.0001 |
| miR-302d | -1.75 | 0.0005 | miR-32 | -1.72 | 0.0479 | miR-21* | +1.94 | 0.0002 |
| miR-23a | +1.75 | 0.0325 | miR-671-5p | -1.71 | 0.0211 | miR-297b-3p | +1.93 | 0.0010 |
| miR-181c | -1.74 | 0.0015 | miR-571 | -1.69 | 0.0093 | miR-466f-3p | +1.89 | 0.0049 |
| miR-512-5p | -1.72 | 0.0217 | miR-668 | +1.69 | 0.0085 | miR-709 | +1.87 | 0.0025 |
| miR-409-5p | -1.72 | 0.0243 | miR-190 | -1.67 | 0.0064 | miR-199b-5p | -1.86 | 0.0029 |
| miR-879 | -1.72 | 0.0103 | miR-295* | +1.66 | 0.0093 | miR-691 | +1.86 | 0.0009 |
| miR-31 | +1.71 | 0.0007 | miR-21* | +1.66 | 0.0027 | miR-523 | -1.86 | 0.0121 |
| miR-558 | -1.71 | 0.0070 | miR-192 | -1.64 | 0.0222 | miR-302d* | +1.83 | 0.0046 |
| miR-141 | -1.70 | 0.0024 | miR-17-3p | -1.63 | 0.0033 | miR-302d | -1.83 | 0.0002 |
| miR-92a-2* | -1.69 | 0.0097 | miR-24-2* | +1.63 | 0.0139 | miR-665 | +1.82 | 0.0019 |
| miR-93 | -1.69 | 0.0004 | miR-330 | -1.63 | 0.0267 | miR-503 | +1.79 | 0.0046 |
| miR-376c | -1.68 | 0.0258 | miR-941 | -1.62 | 0.0240 | miR-149* | +1.79 | 0.0061 |
| miR-296* | -1.65 | 0.0072 | miR-18b* | -1.62 | 0.0046 | miR-193a-5p | +1.77 | 0.0075 |
| hsa-miR-541* | -1.65 | 0.0034 | miR-330-3p | -1.61 | 0.0260 | miR-887 | +1.77 | 0.0013 |
| miR-297b-3p | +1.64 | 0.0083 | miR-362-5p | -1.60 | 0.0042 | miR-31 | +1.75 | 0.0004 |
| let-7e | +1.63 | 0.0221 | miR-465c-3p | +1.59 | 0.0274 | miR-663 | -1.74 | 0.0013 |
| miR-362-5p | -1.63 | 0.0032 | miR-92a-2* | -1.59 | 0.0197 | miR-558 | -1.72 | 0.0114 |
| miR-335 | -1.61 | 0.0022 | miR-297c* | +1.58 | 0.0136 | miR-675-5p | +1.71 | 0.0080 |
| miR-302c | -1.60 | 0.0376 | miR-933 | +1.58 | 0.0256 | miR-200b* | +1.69 | 0.0068 |
| miR-27b* | -1.60 | 0.0066 | miR-587 | -1.58 | 0.0050 | miR-290 | +1.67 | 0.0069 |
| miR-224 | +1.58 | 0.0091 | miR-545 | -1.57 | 0.0039 | miR-487b | +1.67 | 0.0048 |
| miR-673 | -1.57 | 0.0421 | miR-879 | -1.57 | 0.0279 | miR-335 | -1.67 | 0.0012 |
| miR-370 | -1.57 | 0.0231 | miR-302b | -1.57 | 0.0083 | miR-369-5p | -1.66 | 0.0182 |
| miR-758 | -1.57 | 0.0322 | miR-15b* | -1.56 | 0.0055 | miR-539 | -1.66 | 0.0152 |
| miR-21* | +1.56 | 0.0065 | miR-879* | -1.55 | 0.0246 | miR-135a* | +1.66 | 0.0078 |
| miR-32* | +1.56 | 0.0126 | miR-887 | +1.54 | 0.0096 | miR-507 | -1.66 | 0.0034 |
| miR-874 | -1.56 | 0.0248 | miR-877 | -1.54 | 0.0022 | miR-685 | +1.66 | 0.0151 |
| miR-191* | -1.56 | 0.0442 | miR-541* | -1.54 | 0.0094 | miR-433* | +1.66 | 0.0183 |
| miR-485 | -1.56 | 0.0042 | miR-620 | +1.52 | 0.0162 | miR-15b* | -1.65 | 0.0023 |
| miR-684 | -1.56 | 0.0108 | miR-376c* | -1.52 | 0.0290 | miR-549 | +1.65 | 0.0108 |
| miR-708* | -1.56 | 0.0204 | miR-522 | -1.52 | 0.0109 | miR-19b-1* | -1.65 | 0.0207 |
| miR-602 | -1.55 | 0.0088 | miR-421 | -1.52 | 0.0206 | miR-939 | +1.64 | 0.0086 |
| miR-492 | -1.55 | 0.0500 | miR-602 | -1.52 | 0,0124 | miR-498 | +1.64 | 0.0074 |
| miR-493 | -1.55 | 0.0016 | miR-650 | -1.52 | 0.0161 | miR-589* | -1.64 | 0.0072 |
| miR-15b* | -1.54 | 0.0070 | miR-224 | +1.52 | 0.0162 | miR-620 | +1.64 | 0.0061 |
| miR-587 | -1.54 | 0.0076 | miR-560 | -1.51 | 0.0130 | miR-466d-3p | +1.63 | 0.0034 |
| miR-629* | -1.54 | 0.0141 | miR-335 | -1.51 | 0.0060 | miR-690 | +1.63 | 0.0078 |
| miR-371-5p | +1.53 | 0.0469 | miR-559 | -1.51 | 0.0061 | miR-32* | +1.62 | 0.0076 |
